# Time-Dependent Particle-Breaking Hartree–Fock Model for Electronically Open Molecules

**DOI:** 10.1021/acs.jpca.5c00810

**Published:** 2025-05-01

**Authors:** Jacob Pedersen, Bendik Støa Sannes, Regina Paul née Matveeva, Sonia Coriani, Ida-Marie Høyvik

**Affiliations:** † Department of Chemistry, Technical University of Denmark, DK-2800 Kongens Lyngby, Denmark; ‡ Department of Chemistry, Norwegian University of Science and Technology, N-7491 Trondheim, Norway

## Abstract

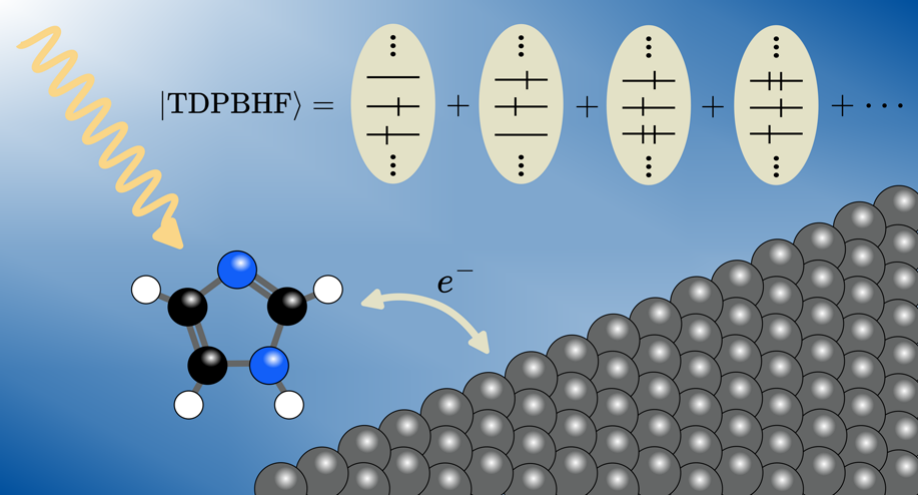

We develop the time-dependent particle-breaking Hartree–Fock (TDPBHF) model to describe excited states and linear response properties of electronically open molecules. This work represents the first step toward building a wave function-based response theory framework for electronically open quantum systems equivalent to that of closed quantum systems. In the limit of particle conservation, TDPBHF reduces to standard time-dependent Hartree–Fock theory. We illustrate the TDPBHF model by computing valence absorption spectra and frequency-dependent electric dipole polarizabilities for a set of small- to medium-sized organic molecules. The particle-breaking interactions are observed to nonuniformly redshift the excitation energies and induce qualitative changes in the absorption profile. In addition, the mixing of multiple excitations in the TDPBHF wave function is observed to dampen the divergence of the response function in the vicinity of resonance energies.

## 1 Introduction

Historically, electronic wave function theory has been built on closed quantum system wave function parametrizations.^1^ A closed quantum system does not exchange information (energy and/or particles) with its environment. In contrast, open quantum systems are characterized by being coupled to some environmental degrees of freedom.^2−5^ To allow for interfacial charge and energy exchange, the quantum system of interest and the environment must be able to interact.^6^ It has become standard practice to model the environment and its equilibrium effect (that being intermolecular energy fluctuations, i.e., nonzero energy spread) on the quantum system of interest through the use of effective Hamiltonians, as done, e.g., in quantum embedding^7−19^ and multilevel^20−34^ models. The explicit introduction of an interacting environment in the wave function model for the target quantum system amounts to considering an energetically open quantum system.^35^ Despite that, the inclusion of interfacial electron fluctuations, and thus the concept of electronically open quantum systems, have been largely overlooked in the context of wave function theory. We are interested in describing electronically open molecules due to noncovalent equilibrium interactions with environments such as solvent molecules or physisorbing molecular surfaces. Meanwhile, we are not interested in a full quantum-mechanical description of the environment, but solely in the effects of intermolecular charge fluctuations on the molecular system.

In this work, we will make use of an effective Hamiltonian. The primary advantage of the effective Hamiltonian approach is the continued use of electronic wave functions and the ease with which existing computational protocols from standard electronic structure theory can be adopted and applied. In open quantum system theory, it is customary to partition the Hamiltonian of the composite system (i.e., molecule and environment) into an isolated molecule, an environment, and an interaction Hamiltonian accounting for their coupling.^2,3^ When working in a basis of localized spin orbitals assigned to either the molecule or the environment, the interaction Hamiltonian can be divided into a component responsible for the exchange of energy (while conserving the electron number of the molecule) and one that permits particle fluctuations between molecule and environment.^36−38^ The particle-conserving interactions can be wrapped into the Hamiltonian for the molecule as an effective interaction operator, as is done in various quantum embedding^7−19^ and multilevel^20−34^ schemes. The second component of the interaction Hamiltonian does not conserve the number of electrons, and these terms are thus characterized as particle-breaking.^36^ The particle-breaking interactions are expected to be small compared to the particle-conserving interactions. In this work, we consider only the particle-breaking interactions. The particle-conserving interactions may be included using existing embedding or multilevel models.

The construction of effective Hamiltonians capable of facilitating charge fluctuations between the molecule and the environment necessitates particle-breaking Hamiltonians and thus electronic wave functions that are not eigenfunctions of the number operator.^36^ In a related, but different, category are models allowing transitions between states of different integer numbers of electrons. This includes electron-attachment and electron-detachment equation of motion coupled cluster methods.^39−43^ These methods utilize effective creation and annihilation operators to add or remove electrons,^44^ and they can be used to describe spectroscopies,^45,46^ such as Auger-Meitner electron spectroscopy.^47−49^ A particularly interesting (with respect to electronic openness) type of state encountered in such spectroscopies is electronic resonances, which are metastable bound states coupled to the continuum.^50^ The continuum component in the wave function makes the electronic resonances electronically open from a bound state perspective. Meanwhile, the open boundary conditions of the continuum cannot be described with the square-integrable (*L*^2^) basis functions typically used in standard electronic structure programs. This can be circumvented, for example, by recasting the problem in terms of non-Hermitian quantum mechanics.^50,51^ One example is the inclusion of a complex absorbing potential in the Hamiltonian.^52,53^ The use of complex absorbing potentials thus represents another example of using effective Hamiltonians for wave function models to describe open quantum systems. Other non-Hermitian approaches to open quantum systems include the effective Hamiltonian methods that map master equations into Schrödinger-like time evolution equations and include dissipation effects through the use of effective non-Hermitian Hamiltonians.^54,55^

In comparison to the use of an effective Hamiltonian to account for environmental interactions, the more traditional approaches in open quantum system theory for describing energetically open quantum systems are based on the density operator formalism.^2−5^ This alternative choice is particularly convenient when considering architectures such as molecular junctions (i.e., molecules sandwiched between electrodes).^56−58^ The major difference between modeling molecules in molecular junctions versus molecules in solutions or physisorbed onto molecular surfaces is the nonequilibrium conditions enforceable through the application of a bias voltage across the molecular junction. Decoherence processes such as dissipation and dephasing become important in nonequilibrium situations, and these cannot easily be attained with wave function theory.^59,60^ Nonetheless, an example of wave function theory capable of accounting for dephasing is the method recently presented by Coccia et al. based on Markovian stochastic Schrödinger equations.^61^ Typically, the more general and flexible density operator formalism is adopted, and the use of reduced description quantum master equations becomes more convenient for parametrizing the environmental interaction.^2−5^ Briefly, these methods are based on statistical averaging over the environmental degrees of freedom and subsequent development of time evolution equations for the resulting reduced density representing only the molecule, with the environmental effects included implicitly, thereby making it electronically open.^2,3,5^ The advantages of such methods include charge and energy transfer (and thus the concept of finite lifetimes) to naturally arise from the interaction between the molecule and the environment. In contrast, phenomenological dampening must be introduced to account for the finite lifetimes of excited states in standard electronic structure theory.^62,63^ On the downside, reduced description quantum master equations come with the cost of immense complexity, which significantly limits their applicability.^2,3,5^

Recently, we presented the restricted and unrestricted particle-breaking Hartree–Fock (PBHF) model for electronically open molecules.^36,37^ This marks the beginning of building a framework of correlated wave function and response theory methodologies for electronically open quantum systems analogous to that of closed quantum systems. The PBHF model is a mean-field theory and parametrizes the fluctuations of electrons in the molecule due to its equilibrium interaction with the environment through an effective particle-breaking Hamiltonian. The PBHF Hamiltonian necessitates a wave function that breaks the particle-number symmetry, which in PBHF theory is achieved through a combination of determinants with different numbers of electrons. This combination of determinants with different particle-numbers gives rise to fractional charging, due to electron fluctuations between the molecule and the environment.^36^

The concept of fractional orbital occupancy has been used to recover static correlation effects in single-determinantal wave functions as an alternative to multi-determinantal wave function approaches. Examples include grand canonical Hartree–Fock (GCHF) methods,^64−67^ ensemble density functional theory (DFT),^68−74^ and the Hartree–Fock–Bogoliubov (HFB) method.^75,76^ From the viewpoint of our PBHF model, a closely related parametrization of the density operator (based on ensemble DFT) was proposed by Nygaard and Olsen.^73^ The main difference between their ensemble DFT model and our PBHF model is that the former does not consider the particle-breaking interactions. Hence, their energy expression does not include the pairing energy contribution found in our PBHF model.^36^ In a related context, non-integer electron numbers are central for justifying the mathematical existence of particle-number-related reactivity descriptors (i.e., partial derivatives) in conceptual DFT. Miranda-Quintana and Ayers have recently proposed that fractional electron numbers naturally originate from viewing reactants as open quantum systems interacting with their environment.^77^

The HFB method (which merges the Hartree–Fock (HF) mean-field description with the electron-pairing correlation from Bardeen–Cooper–Schrieffer (BCS) theory^78^) is closely related to our PBHF model, and we expand on this in the following. Specifically, the HFB method is based on a Bogoliubov transformation of the fermionic creation and annihilation operators into isomorphic quasi-particle operators, hereby making the HFB wave function represent a quasi-particle vacuum.^79^ The PBHF wave function parametrization is equivalent to filling an HFB state with quasi-particles, such that the expansion point (and dominant configuration) is the HF wave function. The filling of quasi-particles amounts to a rotation of the HFB wave function by π/2 in the Fock space spanned by the occupation angles of the standard HF-occupied orbitals. The different choices of starting points for the wave function expansions in PBHF and HFB are motivated by their different physical origins and purposes. As previously mentioned, the objective of HFB is to recover static correlation, while PBHF aims to describe small fluctuations in the number of electrons around a single reference determinant due to environmental interactions. The electron fluctuations are due to dynamical correlation effects between the considered molecule and its (implicitly described) environment. Consequently, the PBHF wave function must be expanded around a single determinant, whereas no such requirement is imposed on the HFB wave function. The PBHF model is thus motivated by its physical origin (particle-breaking terms in the Hamiltonian for the molecule interacting with its environment) and the required wave function expansion point (Hartree–Fock).

The purpose of this work is to develop time-dependent particle-breaking Hartree–Fock (TDPBHF) theory to enable investigations of the effects of electronic openness on excited electronic manifolds and response properties. We restrict our model to consider only the time dependence of the orbitals, whereas the occupation angles are assumed to be time-independent. In other words, we include only local excitations (i.e., excitations within the molecule). This choice is motivated by the notion that the inclusion of nonlocal excitations (i.e., excitations leading to electron transfer to and from the environment) necessitates the introduction of explicit environmental relaxation, which contravenes the purpose of using an effective Hamiltonian for the environmental interaction. Thereby, our work represents the first step toward building a wave function-based response theory framework for electronically open quantum systems equivalent to that of closed quantum systems. This interest stems from both a purely fundamental perspective in understanding the mechanisms of interfacial charge and energy fluctuations, and an applied perspective in designing novel materials with electronic properties tailored to specific applications.

In the following, we will adopt the restricted formulation of PBHF,^36^ and our work is heavily inspired by the response treatment presented in refs (80 and 81). Specifically, we will resort to the frequency-domain formulation of time-dependent response theory since we target excitation energies and transition moments which play central roles in this formulation.^80−82^ In addition, we are interested in the frequency-dependent electric dipole polarizability since it represents a very suitable response property for probing charge distributions and thus electronic openness. In the quasi-energy formulation of response theory, response properties are encoded in the response functions, defined as derivatives of the time-averaged quasi-energy, through the generalized Hellman–Feynmann theorem.^81^ Besides, transition and excited state properties can be obtained as residues of the response functions.^80,81,83^

As previously mentioned, the particle-conserving interactions may be included using existing models. The fluctuating charge (FQ) model^84^ would be natural to use in combination with our particle-breaking framework. Specifically, the FQ model is based on the principle of electronegativity equalization,^85,86^ stating that each atom in the molecule has the same electronegativity at equilibrium.^87^ It is motivated by the notion of charge transfer between atomic sites with different electronegativity in nonequilibrium situations, and parametrizes the problem by considering the atomic charges as variables that can change in response to the environment. In other words, the FQ model allows charge transfer redistribution within the environment, but the molecule under consideration remains electronically closed.^87,88^ The combination with our particle-breaking framework is thus the missing component required to account for the electronic openness of the molecule.

TDPBHF theory reduces to standard time-dependent Hartree–Fock (TDHF) theory in the limit of particle conservation. Therefore, TDPBHF represents the generalization of TDHF to electronically open quantum systems. TDHF is in the nuclear physics community known as the particle-hole random phase approximation (ph-RPA) and is often used to recover dynamic correlation in single-determinantal wave functions.^79,89,90^ Additionally, particle–particle RPA (pp-RPA) has been developed to include additional correlation effects through the use of two-body creation operators in the occupied and virtual space.^91^ The ph- and pp-RPA channels have been collected in the quasi-particle RPA (qp-RPA) formulation which is rooted in the HFB method.^91^ However, the purpose of qp-RPA is to recover dynamic correlation for the electronic ground state, while the objective of standard TDHF in the quantum chemistry community has been to target excited states and response properties,^92^ as is the purpose of the TDPBHF model.

The paper is organized as follows. First, the PBHF model is outlined and the TDPBHF response equations are derived in Section 2. To illustrate our method, we compute valence absorption spectra and frequency-dependent electric dipole polarizabilities. The computational details are provided in Section 3, and we discuss the results in Section 4. Lastly, we give a conclusion and outlook in Section 5.

## 2 Theory

In this section, we present the TDPBHF model. The section starts with a brief overview of the ground state PBHF model before the standard response equations are outlined and the TDPBHF working equations are derived. Lastly, we provide the equations for the investigated transition properties and electric dipole polarizability.

### 2.1 Particle-Breaking Hartree–Fock Model

In the PBHF model, the interaction between the molecule and the environment is parametrized by means of the particle-breaking Hamiltonian
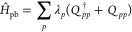
1with λ_*p*_ being some empirical parameters representing the strength of the environmental interaction. *Q*_*pp*_^†^ and *Q*_*pp*_ are the closed-shell pair creation and annihilation operators, respectively
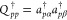
2
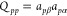
3where *a*_*p*σ_^†^ and *a*_*p*σ_ are the fermionic creation and annihilation operators, respectively. Thereby, the particle-breaking Hamiltonian can create and/or annihilate pairs of electrons in the molecule. This form of the particle-breaking Hamiltonian was introduced in ref (36) through consideration of the particle-breaking two-electron interactions occurring between electrons from a quantum system of interest and an environment upon averaging out the environmental electronic degrees of freedom.

The total effective Hamiltonian  is constructed by adding the particle-breaking Hamiltonian to the standard molecular electronic Hamiltonian _mol_
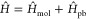
4We note that we have omitted the particle-conserving interactions, which may be included by using existing quantum embedding and multiscale models. The molecular electronic Hamiltonian (excluding nuclear repulsion) in second quantization form reads
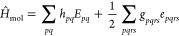
5with the one- and two-electron integrals (in atomic units)
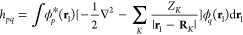
6
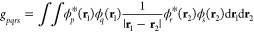
7where **r**_*n*_ is the position vector of the *n*th electron, **R**_*K*_ is the position vector of the *K*th nucleus with charge *Z*_*K*_, and {ϕ_*p*_} represents the set of real molecular orbitals. *E*_*pq*_ and *e*_*pqrs*_ are the singlet one- and two-electron excitation operators
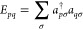
8
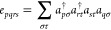
9

The Hamiltonian in eq 4 does not commute with the molecule number operator *N̂* = ∑_*p*_*E*_*pp*_ due to _pb_. The wave function for this Hamiltonian should therefore not be an eigenfunction of *N̂*, but rather include states with different numbers of electrons. In the PBHF model, such state is generated through a unitary transformation of an *N*-electron closed-shell reference determinant |Φ⟩. Specifically,
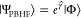
10where exp(γ̂) is a unitary operator generated by the anti-Hermitian operator
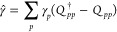
11The γ_*p*_ parameters are called occupation angles due to their connection to the one-electron density matrix,
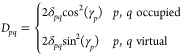
12where occupied and virtual indices refer to the orbitals of the *N*-electron reference determinant. In addition, the PBHF energy requires the definition of the pairing density matrix,
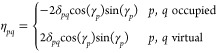
13The PBHF wave function is variationally optimized with respect to both the occupation angles and the orbitals. For more details on PBHF, see ref (36).

We now show that the PBHF wave function is equivalent to an HFB state filled with quasi-particles. In the following, we adopt the standard orbital index notation, where *i*, *j*, ··· refer to occupied orbitals and *a*, *b*, ··· denote virtual orbitals (with respect to the *N*-electron reference determinant), and write the PBHF wave function as
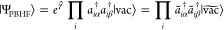
14where *ã*_*i*σ_^†^ = exp(γ̂)*a*_*i*σ_^†^exp(−γ̂) are the Bogoliubov-transformed creation operators.  is noted to be the quasi-particle vacuum, as it fulfills the killer condition^93^
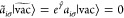
15Hence, eq 14 shows that the PBHF wave function is an HFB state filled with quasi-particles. This results in single-particle occupancies in the quasi-particle state.^93^ In the PBHF model, we create quasi-particles in all the standard HF-occupied orbitals; therefore, the resulting state will be largely dominated by the HF determinant.

A geometrical interpretation of the PBHF wave function in terms of the HFB wave function can be obtained by noting that the Bogoliubov transformation in the PBHF model entails orbital occupations following cos^2^(γ_*i*_) and sin^2^(γ_*a*_) in the occupied and virtual space of the quasi-particle state, respectively. Thus, by rotating the occupied orbitals (i.e., shifting the occupation angles of the standard HF-occupied orbitals) in the PBHF wave function by π/2, we recover the HFB wave function.

As the PBHF wave function is expanded around the HF determinant, it will stay close to that configuration for physical values of the environment interaction. For a molecule noncovalently bound with an environment this is approximately in the order of 10^–4^–10^–1^. Large values of the λ_*p*_ parameters, on the other hand, would be relevant for orbitals ϕ_*p*_ that share an electron pair equally with the environment, e.g., a covalent bonding situation. The overlap between the HF determinant and the PBHF wave function evaluates (derivation provided in Section S1 in the Supporting Information (SI)) to
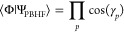
16In the limit of particle conservation, all occupation angles are zero and the overlap is 1.0. The overlap between the HF and the PBHF wave functions measures the amount of HF character in the PBHF wave function. The amount of HF character depends on the degree of electron fluctuations between the molecule and the environment. Hence, the overlap offers a convenient measure of the electronic openness of the molecule. The electronic openness induces fractional charging of the molecule, i.e., the molecule acquires a fractional charge. The fractional charging is quantified by
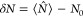
17with *N*_0_ being the number of electrons in the electronically closed molecule.

### 2.2 Time-Dependent Particle-Breaking Hartree–Fock

Based on the restriction to local excitations (i.e., excitations wholly within the molecule), we assume that the occupation angles are time-independent. Hence, we write the TDPBHF wave function as
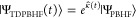
18where κ̂(*t*) is the time-dependent orbital rotation operator
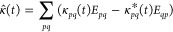
19with κ_*pq*_(*t*) being the time-dependent orbital rotation parameters. In this way, the TDPBHF wave function resembles the standard TDHF wave function. It may therefore be shown that the formal derivation and existing computational protocols are directly applicable, with the only difference being the PBHF wave function instead of the HF wave function.

For a variational exponential wave function parametrization, this results in the first-order response equation (commonly called the standard response equation^94^)
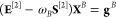
20where **E**^[2]^ and **S**^[2]^ are the Hessian and metric matrix, respectively. **X**^*B*^ is the response vector which amounts to the collection of first-order (linear) response parameters, and **g**^*B*^ is the property gradient vector that describes the coupling between the incoming perturbing field and the molecule (with *B̂* and ω_*B*_ being the perturbation operator and optical frequency of the field, respectively). The Hessian, metric, and property gradient take the form^81,83^
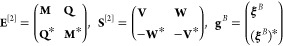
21with the matrix and vector elements
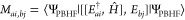
22
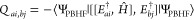
23
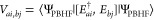
24
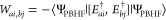
25
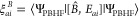
26The standard response equation (eq 20) is solved using an iterative subspace method.^95^ In the absence of an external perturbation, the property gradient **g**^*B*^ in eq 20 vanishes and the response equation reduces to the generalized eigenvalue problem
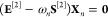
27where **X**_*n*_ now contain the amplitudes of the transitions between the ground state and *n*th excited state, with ω_*n*_ = *E*_*n*_ – *E*_0_ being the corresponding transition frequencies. Equation 27 is commonly called the response eigenvalue equation.^94^

As previously mentioned, the action of exp(γ̂) on the *N*-electron reference wave function generates a linear combination of states with different numbers of electrons. This complicates the evaluation of expectation values, since it requires keeping track of electrons between Fock spaces of different dimensionality, but also because the concept of occupied and virtual orbitals becomes ambiguous due to the possibility of fractional orbital occupations. Therefore, it is much more convenient to work with the Bogoliubov-transformed operators in comparison to working directly with the PBHF wave function. The Bogoliubov transformation of the operators comes from unpacking the PBHF wave function
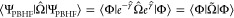
28where we have defined the transformation of the operator Ω̂ through the last equality.

Consequently, to obtain explicit expressions for the TDPBHF matrix and vector elements in eqs 22–26, we first evaluate the commutators (provided in Section S2 in the SI), and then unpack the PBHF wave function and Bogoliubov-transform the operators. The matrix elements in eqs 22–25 take the form
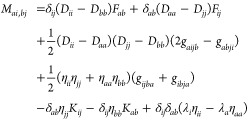
29
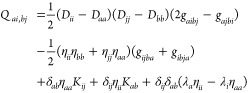
30
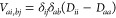
31
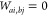
32The density matrix and pairing matrix are defined in eqs 12 and 13, respectively. The Fock matrix elements, *F*_*pq*_, and the exchange matrix elements, *K*_*pq*_, are defined by
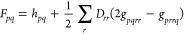
33
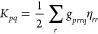
34Note that the Fock matrix is not diagonal in the PBHF model, since we use density-based energy optimization rather than Fock matrix diagonalization.

For a one-electron operator *B̂* = ∑_*pq*_*B*_*pq*_*E*_*pq*_, with *B*_*pq*_ = ⟨ϕ_*p*_|*B̂*|ϕ_*q*_⟩ being the one-electron integral, the property gradient is given by
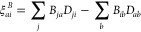
35Lastly, we note that the PBHF wave function reduces to the standard HF wave function in the particle conserving limit (i.e., lim_λ→0_|Ψ_PBHF_⟩ = |Φ⟩), in which the standard TDHF matrix and vector expressions are recovered. TDPBHF thus represents the generalization of TDHF to electronically open quantum systems, with standard TDHF being the limiting case of an electronically closed quantum system.

### 2.3 Response Properties

The electric dipole polarizability is identified as the negative linear response function
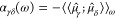
36with μ̂_γ_ being the Cartesian component γ of the electric dipole moment operator
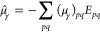
37The linear response function is computed by contraction of the property gradient (eq 21) and response vector (eq 20) employing the electric dipole moment operators
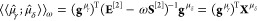
38The isotropic electric dipole polarizability is given by
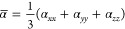
39

The TDPBHF excitation energies are calculated as poles of the linear response function as obtained by solving the response eigenvalue problem in eq 20. Meanwhile, we found it convenient to reformulate (derivation provided in Section S3 in the SI) the eigenvalue equation to the form of Casida,^96^
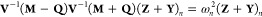
40where we have partitioned the amplitude vector **X**_*n*_ into an excitation vector **Z**_*n*_ and deexcitation vector **Y**_*n*_. We note that **V** is diagonal, and the computational cost of the matrix inversion appearing on the left-hand side of the equation is thus negligible. Equation 40 is solved iteratively, similar to the standard response equation (eq 20).

The residues of the linear response function at the excitation energies give the transition moments^81^*T*_μ_γ__^0→*n*^,
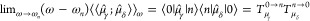
41The transition moments are computed by contracting the property gradient (eq 21) and excitation amplitude vector (eq 27)
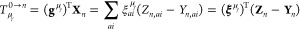
42The transition moments are related to the one-photon absorption oscillator strength *f*_osc_ in length gauge by
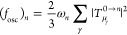
43

## 3 Computational Details

**Figure 1:**
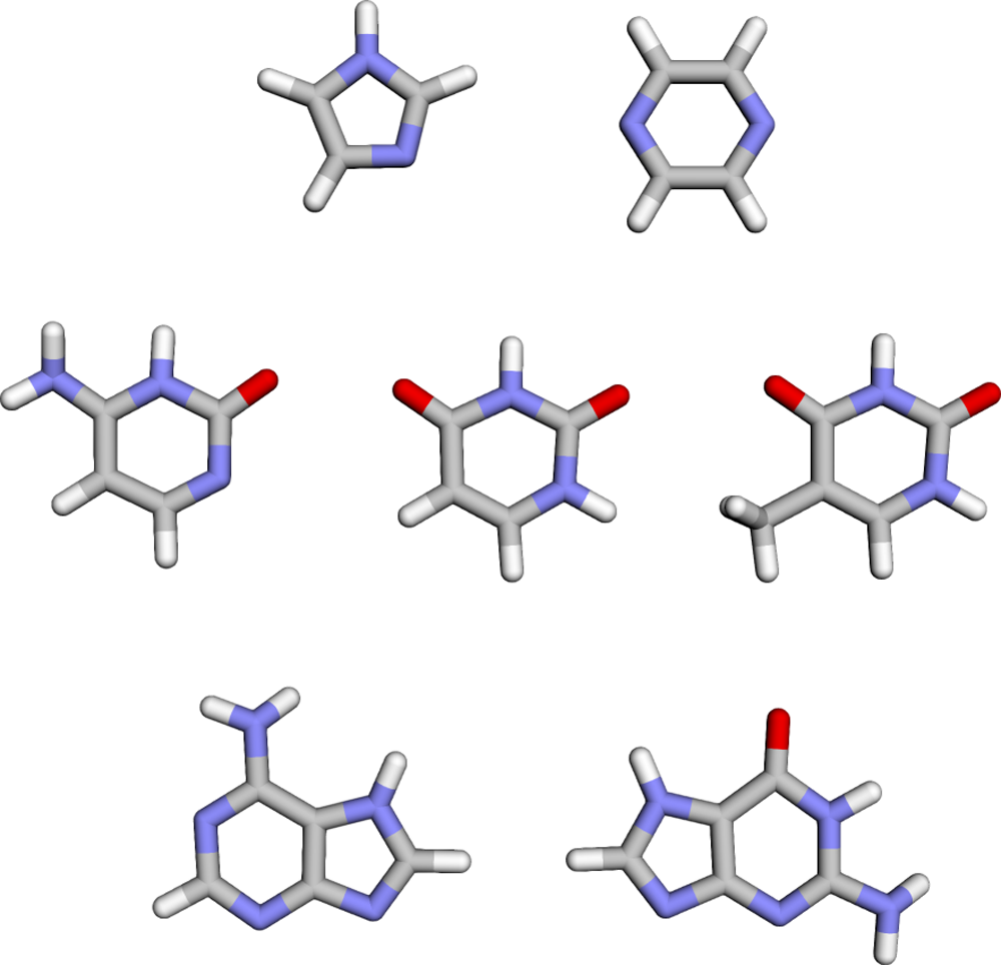
Structures of (left to right, top to bottom) imidazole, pyrazine, cytosine, uracil, thymine, adenine, and guanine.

The TDPBHF method has been implemented in a development version of the *e*^*T*^ electronic structure program.^97^ We provide numerical illustrations of the developed methodology for the molecules imidazole, pyrazine, cytosine, uracil, thymine, adenine, and guanine (Figure 1). They are selected based on their particularly rich valence absorption spectra, which makes them excellent candidates for investigating the effects of electronic openness on electronic excited state manifolds. Their structures were optimized at the MP2/cc-pVTZ level of theory in Gaussian 16 (Rev. A.03)^98^ with default convergence thresholds. All TDHF and TDPBHF calculations have been performed utilizing the aug-cc-pVDZ and cc-pVDZ basis sets^99,100^ for non-hydrogenic and hydrogen atoms, respectively. For the TDPBHF computations, we formed active spaces (specified in Section S4 in the SI) consisting of reference wave function orbitals within the energy interval [(ε_HOMO_ – λ_max_); (ε_LUMO_ + λ_max_)], with ε_HOMO_ and ε_LUMO_ being the orbital energy of the highest occupied molecular orbital (HOMO) and lowest unoccupied molecular orbital (LUMO), respectively, and λ_max_ = 0.1 a.u. being the largest interaction strength applied. The active orbitals were coupled uniformly, i.e., we used the same environmental interaction strength for all the active orbitals, and we performed calculations using λ = *k* · 0.01 a.u. for *k* = 1, 2, ···, 10.

The valence absorption spectra were constructed by computing the first 50 excited states and broadening the spectra with Gaussian functions with a standard deviation of 0.1 eV. We note that our computations are performed without symmetry. Therefore, to keep track of the excited states, we label the transitions a posteriori by considering their energetic placement, magnitude and dipole direction of the corresponding transition strengths (eq 41) according to the standard selection rules, and by analysis of the excitation amplitudes. This rudimentary labeling protocol is sufficient for the purpose of this work. The electric dipole polarizabilities are computed at the frequencies ω = *k* · 0.01 a.u. for *k* = 0, 1, ···, *k*_max_, where *k*_max_ is the greatest integer such that ω ≤ ω_res_ with ω_res_ being the first resonance energy.

## 4 Results and Discussion

We now present the effects of the particle-breaking interactions on the investigated properties. The section is organized as follows. First, we illustrate the effects of the environmental interaction on the valence absorption spectra, i.e., the transition properties. Next, we consider the dispersion of the frequency-dependent electric dipole polarizability as well as the static polarizability with respect to the environmental interaction. The results computed with standard TDHF (i.e., λ = 0) are noted to correspond to the limit of electronically closed molecules.

### 4.1 Valence Absorption Spectra

**Figure 2:**
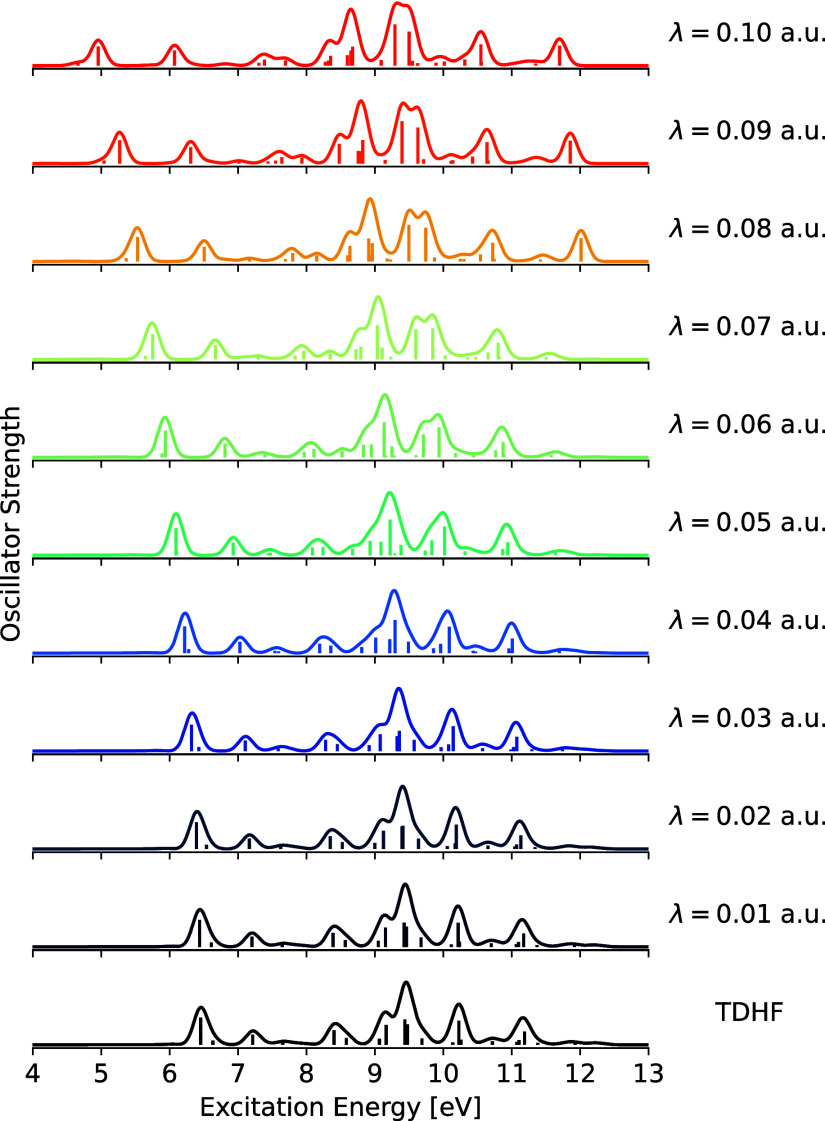
Valence absorption spectrum of imidazole computed with different interaction strengths. Each spectrum is constructed from 50 excitations by broadening with Gaussian functions with a standard deviation of 0.1 eV. Only sticks corresponding to *f*_osc_ ≥ 0.01 are visualized.

In Figure 2 we present the absorption spectrum of imidazole computed with different interaction strengths. The observed shifts and qualitative changes in the absorption spectra for imidazole are representative for all molecules considered. For brevity, we therefore present results for the other molecules in Section S5 in the SI. To aid in the interpretation of the observed changes in the absorption profile of imidazole, we have provided a 3D plot in Figure S1 in the SI.

Before discussing the results, we note that state-labeling is mandatory for keeping track of the transitions, as the environmental interaction induces energetic reordering of the excited states (relative to the reference electronically closed molecule). For example, such energetic reordering is observed between the low-lying transitions 1A′ → 2A′ and 1A′ → 2A″ comprising the first absorption feature when increasing the interaction strength from λ = 0.05 a.u. to λ = 0.06 a.u. This is illustrated in Figure S2 in the SI, where the evolution of the computed sticks corresponding to the four lowest (including the two aforementioned) transitions is depicted. Similarly, the abrupt rise of an additional feature around 12 eV (with respect to the electronically closed molecule) in the absorption spectra with λ ≥ 0.08 a.u. can be attributed to such excited-state reordering, in which a bright transition is moved down in energy and now captured within the first 50 transitions. Energetic reordering of excited states is also encountered in solvated systems when the environmental interactions are taken into account (i.e., by treating the system as an energetically open molecule).^101,102^

#### 4.1.1 Excitation Energies

Figure 2 reveals that the observed redshift of the excitation energies is nonuniform, i.e., the environmental interaction affects the individual transitions differently. For example, the excitation energies of the bright transitions responsible for the first and last absorption feature (with respect to the electronically closed molecule) for the electronically open molecule computed with the largest interaction strength are redshifted relative to that computed for the reference electronically closed molecule by 1.50 and 0.64 eV, respectively. That is, the effect of the particle-breaking interactions is more pronounced in the low-lying transitions. Furthermore, the magnitude of the redshift notably intensifies with increasing interaction strength for the lowest-lying transitions, whereas no such effect is observed for the highest-lying transitions. A redshift of a few tenths of an eV is generally observed upon increasing the interaction strength by Δλ = +0.01 a.u., while the same increment in the interaction strength induces redshifts of almost half an eV for the largest interaction strengths with respect to the lowest-lying transitions.

**Figure 3:**
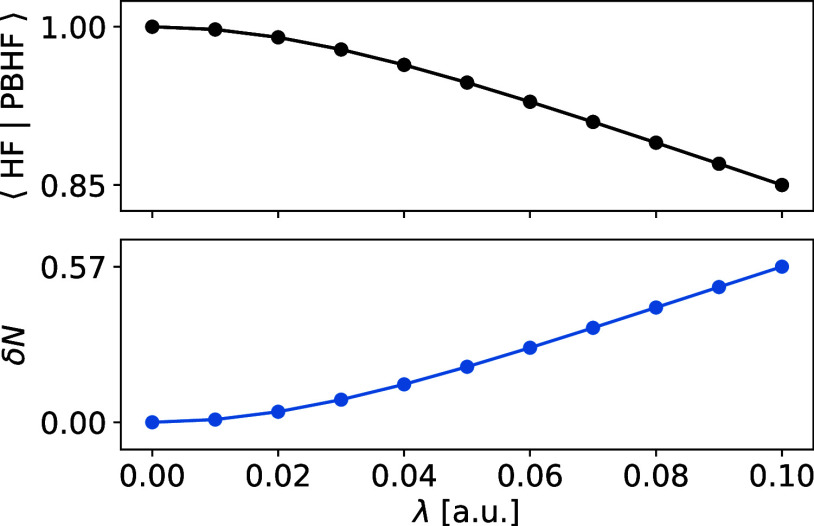
PBHF wave function overlap with HF determinant (top panel) and fractional charging δ*N* with respect to the electronically closed molecule (bottom panel) of imidazole as a function of the environmental interaction strength.

To rationalize the redshift, we present the overlap between the PBHF and HF wave functions (eq 17) computed at different interaction strengths of imidazole in the top panel of Figure 3. The overlap is observed to decrease (i.e., the electronic openness of the molecule increases) with increasing interaction strength. As a brief remark, we note that the wave function of the electronically open molecule computed with the largest interaction strength (λ = 0.1 a.u.) contains 85*%* HF-character, unequivocally demonstrating that the PBHF wave function is largely dominated by the HF determinant as argued in Section 2. The combination of increased electronic openness and an active space of imidazole including mostly virtual molecular orbitals (with respect to the electronically closed molecule) makes the environment effectively act as a source of electrons.^36^ In other words, the environment donates electron density to the molecule, thus resulting in fractional charging of the molecule as depicted in the bottom panel of Figure 3.

The observed redshift of the excitation energies is a direct consequence of the fractional charging. The effect of the fractional charging manifests as a change in the density matrix elements, and thus the prefactors of the Fock matrix elements entering the Hessian (eq 29). In the PBHF model, fractional charging originates from changes in the occupancy of the occupied and virtual molecular orbitals (with respect to the electronically closed molecule). Specifically, whereas *D*_*ii*_^HF^ = 2 and *D*_*aa*_^HF^ = 0 for Hartree–Fock, we have 0 ≤ *D*_*ii*_^PBHF^ ≤ 2 and 0 ≤ *D*_*aa*_^PBHF^ ≤ 2 for PBHF. Hence, the prefactor, δ_*ij*_(*D*_*ii*_^PBHF^ – *D*_*aa*_^PBHF^), multiplied with the virtual-virtual Fock matrix elements in eq 29 is a positive number smaller than the prefactor found in HF (given by δ_*ij*_(*D*_*ii*_^HF^ – *D*_*aa*_^HF^) = 2δ_*ij*_). Similarly, the prefactor for the occupied-occupied Fock matrix elements, δ_*ab*_(*D*_*aa*_^PBHF^ – *D*_*ii*_^PBHF^), is a negative number with smaller magnitude than that for HF (given by δ_*ab*_(*D*_*aa*_^HF^ – *D*_*ii*_^HF^) = −2δ_*ab*_). Hence, considering that the diagonal elements in the virtual-virtual block of the Fock matrix are positive and that the diagonal elements of the occupied-occupied Fock matrix block are negative, we conclude that the relative energy difference between the ground and excited state in PBHF is smaller than the difference between the ground and excited state in HF. This analysis only considers the dominant part and serves only as a crude justification of why a redshift is observed in excitation energies of TDPBHF relative to TDHF.

In other words, the effect of the fractional charging can be viewed as a stabilization of the excited states. The effect of the active space selection on the fractional charging has been investigated in our previous work.^36^ In this work, we have used active spaces as described in Section 3. Consequently, only the low-lying excited states including the active virtual orbitals whose prefactors are affected by the environmental interaction will be stabilized. In contrast, high-lying excited states containing the virtual orbitals excluded in our active space will not experience the same stabilization effect, and this explains the nonuniformity of the observed redshift. Excited-state stabilization effects have similarly been observed in solvated systems, but this effect originates from completely different physics. In particular, solvated-system stabilization effects are a consequence of electrostatic (particle-conserving) interactions between the molecule and environment,^101,102^ whereas the stabilization observed in this work is a consequence of electrons being shared between the molecule and environment.

#### 4.1.2 Oscillator Strengths

As previously mentioned, the particle-breaking interactions induce qualitative changes in the absorption profile. This is a consequence of enhancements and reductions of the oscillator strengths contributing to the absorption features. For example, the absorption feature in Figure 2 just above 10 eV (with respect to the electronically closed molecule) splits into two distinct features around λ = 0.06 a.u. By tracking the excited state, we find that the transition responsible for the splitting of that absorption feature is initially dark and becomes increasingly bright with increasing interaction strength. In contrast, the bright transition corresponding to the first absorption feature gradually loses intensity with increasing interaction strength. The TDPBHF excited state wave functions can (analogously to the ground state wave function) be viewed as some combination of excited states with different numbers of electrons. In the same line of thought, the TDPBHF-computed transitions can be viewed as combinations of excitations in the respective (with respect to electron number) Fock spaces. The observed changes in the oscillator strengths are attributed to this mixing of multiple excitations in the TDPBHF wave function.

Lastly, we comment on the basis set dependence on our findings. The absorption spectrum of imidazole has also been computed (using the same active space) with the aug-cc-pVTZ and cc-pVTZ basis sets^99,100^ for non-hydrogenic and hydrogen atoms, respectively, and the analog of Figure 2 and a comparison plot are provided in Figures S3 and S4 in the SI, respectively. The absorption spectra computed with the triple-ζ basis set combination exhibit some qualitative changes in the bulk part of the absorption profile compared to those computed with the double-ζ basis set combination. Nonetheless, equivalent observations can be made (hence identical conclusions deduced) from the absorption spectra computed with the triple-ζ basis set combination. In this paper, we are only interested in exploring the effects of the particle-breaking interactions, for which the double-ζ basis set results are deemed sufficient. However, as is standard when computing spectroscopic properties, a larger basis set is needed when quantitative calculations are of interest.

### 4.2 Electric Dipole Polarizability

Next, we present the effects of particle-breaking interactions on the electric dipole polarizability. We recall that the response function has poles at frequencies corresponding to the excitation energies, which were found to be redshifted upon increasing the strength of the particle-breaking interactions. This pole movement thus prevents frequency-wise comparison of the electric dipole polarizability at different interaction strengths. Instead, we compare the dispersion of the electric dipole polarizability in the region below the first resonance (which we denote the first resonance region in the following) at different interaction strengths. The particle-breaking interactions are generally found to dampen the divergence of the response function near the pole, i.e., decrease the curvature of the dispersion curve near the pole, with increasing interaction strength. This dampening effect is attributed to the mixing of multiple excitations in the TDPBHF wave function.

**Figure 4:**
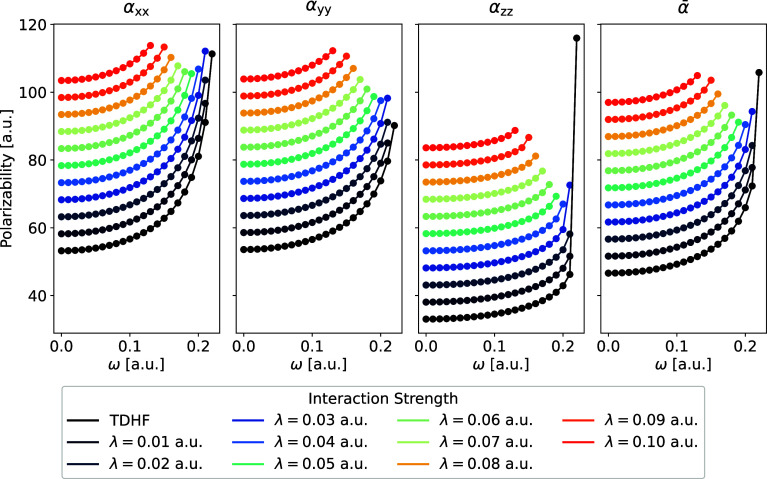
Dispersion curves of the frequency-dependent electric dipole polarizability of imidazole in the first resonance region. Each dispersion curve is vertically offset by 5 a.u. relative to the previous one for comparison purposes.

Figure 4 shows the dispersion of the electric dipole polarizability for imidazole in the first resonance region computed with different interaction strengths. We note that the dispersion curves representing different interaction strengths have been vertically offset by 5 a.u. relative to each other for the sake of comparison. The dampening effect observed in Figure 4 is also illustrated in Figure S6, where we have energetically shifted the dispersion curve for λ = 0.10 a.u. such that the first excitation energy of the electronically open molecule matches that of the reference electronically closed molecule. In general, we observe a similar dampening effect of the response function near the pole for most of the other electronically open molecules. The results of the other molecules are therefore provided in Section S5 in the SI. One exception to the general trend is pyrazine, where little to no dampening is observed near the pole.

To compare directly the effect of the particle-breaking interactions on the electric dipole polarizability (i.e., without the effect of the pole movement), we also investigated the dispersion in the lower half of the first transition region. In this region, the isotropic electric dipole polarizability can be written as a series of even Cauchy moments *S*(−2*n*), in particular, α̅(ω) = ∑_*n*=0_ω^2*n*^*S*(−2*n* – 2), with *n* = 0, 1, ···, *∞*.^103,104^ This is demonstrated for imidazole in Figure S7 in the SI, where an excellent linear regression (*R*^2^ > 0.99) is obtained for all interaction strengths. The estimated Cauchy moments of imidazole are compiled in Table 1 for selected interaction strengths. All estimated *S*(−2) and *S*(−4) Cauchy moments of imidazole are tabulated in Table S1 in the SI. The estimated *S*(−2) Cauchy moment approximates the static isotopic electric dipole polarizability, and the effect of the particle-breaking interactions is observed to be rather small. In contrast, a significant increase is observed for the *S*(−4) Cauchy moment with increasing interaction strength. The product ω^2^*S*(−4) corresponds to the first correction for the dispersion of the electric dipole polarizability, and although the change in *S*(−4) with respect to the interaction strength is quite large, the corresponding contribution to the electric dipole polarizability is very small.

**Table 1:** Estimated Cauchy Moments of Imidazole for Selected Interaction Strengths

λ [a.u.]	0.00	0.02	0.04	0.06	0.08	0.10
*S*(−2) [a.u.]	46.59	46.62	46.71	46.82	46.91	46.98
*S*(−4) [a.u.]	261.64	265.01	274.73	278.15	294.03	322.04

**Figure 5:**
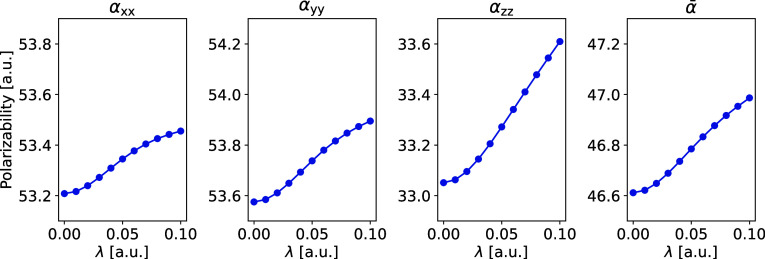
Static electric dipole polarizability of imidazole as a function of the interaction strength λ.

The effect of the particle-breaking interactions is also examined by directly computing the static electric dipole polarizability. In agreement with the findings based on the Cauchy moments, the effect is generally rather small. Figure 5 shows the static electric dipole polarizability of imidazole computed at different interaction strengths, and similar plots for the other molecules are provided in Section S5 in the SI. We note that the *S*(−2) Cauchy moments are almost identical to the directly computed static isotopic electric dipole polarizabilities. The effect of the particle-breaking interactions on the electric dipole polarizability is anticipated to be small. The PBHF model parametrizes small electron fluctuations, and these small fluctuations are not expected to change the molecular charge distribution significantly. Nonetheless, we attribute the observed small changes in the electric dipole polarizability to changes in the spatial extent of the molecular orbitals induced by the particle-breaking interactions.

Lastly, the basis set dependence on our findings related to the electric dipole polarizability was investigated by also carrying out the computations for imidazole with the triple-ζ basis set combination, and virtually no difference compared to the double-ζ results was observed. The results are provided in Section S5 in the SI.

## 5 Conclusions

In this work, we present the TDPBHF model to describe excited states and linear response properties of electronically open molecules. The TDPBHF model extends the ground state PBHF model, which parametrizes the electron fluctuations in molecules induced by noncovalent equilibrium interactions with an electronic environment by means of an effective particle-breaking Hamiltonian. In the limit of particle-conservation, TDPBHF reduces to standard TDHF theory. Consequently, TDPBHF theory is the generalization of standard TDHF to electronically open molecules, and our work represents the first step toward building a wave function-based response theory framework for electronically open quantum systems equivalent to that of closed quantum systems. This is an important stepping stone in our efforts to merge standard wave function-based electronic structure theory with open quantum system theory.

The TDPBHF model is restricted to local excitations, meaning that we consider only the time dependence of the molecular orbitals. Therefore, the derivation of the TDPBHF model is formally equivalent to that of standard TDHF theory with the only difference being the PBHF wave function. The unpacking of the PBHF wave function, i.e., the Bogoliubov transformation of the operators, gives the matrix expressions for the TDPBHF working equations.

We present numerical illustrations of the developed TDPBHF model. Specifically, we compute valence absorption spectra and frequency-dependent electric dipole polarizabilities for a set of small- to medium-sized organic molecules. In general, the particle-breaking interactions are observed to nonuniformly redshift the excitation energies and induce qualitative changes in the absorption profile with increasing interaction strength. The redshift in excitation energies is a consequence of the fractional charging of the molecule induced by the environment. The changes in oscillator strengths are attributed to the mixing of multiple excitations in the TDPBHF wave function.

In terms of the frequency-dependent electric dipole polarizability, the particle-breaking interactions are in general observed to dampen the divergence of the response function near the singularity, i.e., dampen the dispersion curves of the frequency-dependent electric dipole polarizabilities near the resonance energy, with increasing interaction strength. This is similarly attributed to the mixing of multiple excitations in the TDPBHF wave function. To compare directly the effect of the particle-breaking interactions on the electric dipole polarizability for different interaction strengths, we also analyzed the dispersion curve of the polarizability in terms of the leading Cauchy moments *S*(−2) and *S*(−4), and the static electric dipole polarizability (both the individual components and the average value). The effects of the particle-breaking interactions are generally found to be small. This is explained by noting that the PBHF wave function describes small electron fluctuations, which are not expected to significantly affect the molecular charge distribution.

The particle-conserving environmental interactions have been omitted in this work for the purpose of investigating purely the response of fractionally charged systems and the effects of the particle-breaking interactions. Meanwhile, the particle-conserving interactions must be included for a complete description of environmental effects, and we are currently working on combining our particle-breaking model with existing quantum embedding and multiscale methods. As previously mentioned, phenomenological damping must be introduced to account for the finite lifetimes of the excited states induced by the environmental interaction, and this naturally calls for an extension of the presented formalism to damped response theory. We are currently working on such an extension and will present it in future work. Lastly, our particle-breaking formalism should be extended to correlated wave function models.

## Supporting Information Available

Derivation of the overlap between the HF and PBHF wave functions (eq 16); evaluation of the commutators in the *M*_*ai*,*bj*_ and *Q*_*ai*,*bj*_ matrix elements; derivation of eq 40; specification of active spaces; and additional results for imidazole, pyrazine, cytosine, uracil, thymine, adenine, and guanine (PDF)
