# Supplementary material for: Time-Dependent Particle-Breaking Hartree–Fock Model for Electronically Open Molecules
Source: J Phys Chem A. 2025 May 1;129(19):4288–300. doi: 10.1021/acs.jpca.5c00810 (PMC12086847; doi:10.1021/acs.jpca.5c00810)
Supplement: Supplementary file 1 — jp5c00810_si_001.pdf [file jp5c00810_si_001.pdf]

# – Supporting Information –

Time-Dependent Particle-Breaking Hartree-Fock Model for Electronically Open Molecules

Jacob Pedersen[‡,†] Bendik Støa Sannes,[‡] Regina Paul née Matveeva,[‡]

Sonia Coriani,[†] and Ida-Marie Høyvik[*,‡]

†*Department of Chemistry, Technical University of Denmark, DK-2800 Kongens Lyngby, Denmark*

‡*Department of Chemistry, Norwegian University of Science and Technology, N-7491 Trondheim, Norway*

E-mail: ida-marie.hoyvik@ntnu.no

S1 PBHF Overlap

The expression for the overlap between the reference determinant and the PBHF wave function is obtained by

$$\begin{aligned}
\langle \Phi | \Psi_{\mathrm{PBHF}} \rangle &= \langle \Phi | \exp(\hat{\gamma}) | \Phi \rangle \\
&= \langle \Phi | \exp\left(\sum_p \gamma_p (Q_{pp}^\dagger - Q_{pp}) \right) | \Phi \rangle \\
&= \prod_p \langle \Phi | \exp\left(\gamma_p (Q_{pp}^\dagger - Q_{pp}) \right) | \Phi \rangle \\
&= \prod_p \langle \Phi | (1 + \gamma_p (Q_{pp}^\dagger - Q_{pp}) + \frac{1}{2} \left[\gamma_p (Q_{pp}^\dagger - Q_{pp}) \right]^2 + \cdots) | \Phi \rangle \ ,
\end{aligned} \tag{S1}$$

where we have used that $[(Q^\dagger_{pp} - Q_{pp}), (Q^\dagger_{qq} - Q_{qq})] = 0$ to write the third equality. Each expectation value is evaluated separately

$$\gamma_p \langle \Phi | (Q^\dagger_{pp} - Q_{pp}) | \Phi \rangle = 0 \tag{S2}$$

$$\begin{aligned} \frac{1}{2}\gamma_p^2 \langle \Phi | (Q^\dagger_{pp} - Q_{pp})^2 | \Phi \rangle &= \frac{1}{2}\gamma_p^2 \langle \Phi | (Q^\dagger_{pp} Q^\dagger_{pp} - Q^\dagger_{pp} Q_{pp} - Q_{pp} Q^\dagger_{pp} + Q_{pp} Q_{pp}) | \Phi \rangle \\ &= -\frac{1}{2}\gamma_p^2 \langle \Phi | (Q^\dagger_{pp} Q_{pp} + Q_{pp} Q^\dagger_{pp}) | \Phi \rangle \\ &= -\frac{1}{2}\left((\gamma^{\text{occ}}_{pp})^2 + (\gamma^{\text{vir}}_{pp})^2 \right) \\ &= -\frac{1}{2}\gamma_p^2 \ . \end{aligned} \tag{S3}$$

Hence, the overlap becomes

$$\langle \Phi | \Psi_{\text{PBHF}} \rangle = \prod_p (1 - \frac{1}{2}\gamma_p^2 + \cdots) = \prod_p \cos(\gamma_p) \ , \tag{S4}$$

where the cosine series expansion has been identified in the last equality.

S2 Commutators

We find it convenient to evaluate the commutators in the $M_{ai,bj}$ and $Q_{ai,bj}$ matrix elements comprising the Hessian matrix in terms of the standard molecular Hamiltonian and the particle-breaking Hamiltonian separately. The commutator for the standard molecular

Hamiltonian in the $M_{ai,bj}$ matrix element evaluates to

$$\begin{aligned}\big[[E_{ai}^{\dagger}, \hat{H}_{\text{mol}}], E_{bj}\big] = & \, h_{ab}E_{ij} + h_{ij}E_{ba} - \delta_{ab}\sum_{p} h_{pi}E_{pj} - \delta_{ij}\sum_{p} h_{ap}E_{bp} \\ & - \sum_{pqr}(\delta_{ij}g_{aqrp}e_{bqrp} + \delta_{ab}g_{pirq}e_{pjrq}) \\ & + \sum_{pq}(g_{abqp}e_{ijqp} + g_{aqpb}e_{iqpj} + g_{jipq}e_{bapq} + g_{pijq}e_{pabq} - g_{aqjp}e_{iqbp} - g_{piqb}e_{paqj}) \ . \end{aligned} \tag{S5}$$

The commutator for the particle-breaking Hamiltonian in the $M_{ai,bj}$ matrix element yields

$$\big[[E_{ai}^{\dagger}, \hat{H}_{\text{pb}}], E_{bj}\big] = 2(\lambda_i\delta_{ab}Q_{ij} - \lambda_a\delta_{ij}Q_{ab}^{\dagger}) \ . \tag{S6}$$

The commutator for the standard molecular Hamiltonian in the $Q_{ai,bj}$ matrix element becomes

$$\begin{aligned}-\big[[E_{ai}^{\dagger}, \hat{H}_{\text{mol}}], E_{bj}^{\dagger}\big] = & - h_{aj}E_{ib} - h_{ib}E_{ja} - \sum_{pq}(g_{ajqp}e_{ibqp} + g_{aqpj}e_{iqpb} \\ & + g_{bipq}e_{japq} + g_{pibq}e_{pajq} - g_{aqbp}e_{iqjp} - g_{piqj}e_{paqb}) \ . \end{aligned} \tag{S7}$$

The commutator for the particle-breaking Hamiltonian in the $Q_{ai,bj}$ matrix element reads

$$-\big[[E_{ai}^{\dagger}, \hat{H}_{\text{pb}}], E_{bj}^{\dagger}\big] = 2(\lambda_a\delta_{ab}Q_{ij}^{\dagger} - \lambda_i\delta_{ij}Q_{ab}) \ . \tag{S8}$$

Lastly, the following relation is useful for the evaluation of the Bogoliubov-transformed two-electron matrix elements.

$$d_{pqrs} = \langle\Psi_{\text{PBHF}}|e_{pqrs}|\Psi_{\text{PBHF}}\rangle = D_{pq}D_{rs} - \frac{1}{2}D_{qr}D_{ps} + \frac{1}{2}\eta_{pr}\eta_{sq} \ . \tag{S9}$$

S3 Casida Equations

The response eigenvalue equation,

$$(\mathbf{E}^{[2]} - \omega_n \mathbf{S}^{[2]})\mathbf{X}_n = 0 \ , \tag{S10}$$

takes the form

$$\begin{pmatrix} \mathbf{M} & \mathbf{Q} \\ \mathbf{Q}^* & \mathbf{M}^* \end{pmatrix} \begin{pmatrix} \mathbf{Z}_n \\ \mathbf{Y}_n \end{pmatrix} = \omega_n \begin{pmatrix} \mathbf{V} & \mathbf{0} \\ -\mathbf{0} & -\mathbf{V}^* \end{pmatrix} \begin{pmatrix} \mathbf{Z}_n \\ \mathbf{Y}_n \end{pmatrix} \ , \tag{S11}$$

where the amplitude vector $\mathbf{X}_n$ has been split into an excitation vector $\mathbf{Z}_n$ and deexcitation vector $\mathbf{Y}_n$. Alternatively, the response eigenvalue matrix equation can be written as the two coupled equations

$$\mathbf{M}\mathbf{Z}_n + \mathbf{Q}\mathbf{Y}_n = \omega_n \mathbf{V}\mathbf{Z}_n \tag{S12}$$

$$-\mathbf{Q}\mathbf{Z}_n - \mathbf{M}\mathbf{Y}_n = \omega_n \mathbf{V}\mathbf{Y}_n \ , \tag{S13}$$

where we have used that $\mathbf{M}$, $\mathbf{Q}$, and $\mathbf{V}$ are all real matrices to remove the complex conjugation. If we multiply with $(\omega_n \mathbf{V})^{-1}$ from the left on both sides of both equations, we obtain the following equations for the excitation and deexcitation vectors

$$\mathbf{Z}_n = \frac{1}{\omega_n} \mathbf{V}^{-1} (\mathbf{M}\mathbf{Z}_n + \mathbf{Q}\mathbf{Y}_n) \tag{S14}$$

$$\mathbf{Y}_n = -\frac{1}{\omega_n} \mathbf{V}^{-1} (\mathbf{Q}\mathbf{Z}_n + \mathbf{M}\mathbf{Y}_n) \ . \tag{S15}$$

Addition of eqn. (S12) and eqn. (S13) yields

$$(\mathbf{M} - \mathbf{Q})\mathbf{Z}_n + (\mathbf{Q} - \mathbf{M})\mathbf{Y}_n = \omega_n \mathbf{V}(\mathbf{Z}_n + \mathbf{Y}_n) \ . \tag{S16}$$

Substitution of $\mathbf{Z}_n$ and $\mathbf{Y}_n$ results in

$$(\mathbf{M}-\mathbf{Q})\mathbf{V}^{-1}(\mathbf{M}+\mathbf{Q})(\mathbf{Z}+\mathbf{Y})_n = \omega_n^2\mathbf{V}(\mathbf{Z}+\mathbf{Y})_n \ , \tag{S17}$$

where we have written $(\mathbf{Z}_n + \mathbf{Y}_n) = (\mathbf{Z}+\mathbf{Y})_n$. This is a generalized eigenvalue problem with the metric $\mathbf{V}$. To bring it to the form in the manuscript, we move the metric on the right-hand side to the left-hand side of the equation,

$$\mathbf{V}^{-1}(\mathbf{M}-\mathbf{Q})\mathbf{V}^{-1}(\mathbf{M}+\mathbf{Q})(\mathbf{Z}+\mathbf{Y})_n = \omega_n^2(\mathbf{Z}+\mathbf{Y})_n \ . \tag{S18}$$

S4 Active Spaces

The active spaces used in the TDPBHF computations are:
imidazole: [(17-18); (19-28)]
pyrazine: [(19-21); (22-34)]
cytosine: [(28-29); (30-40)]
uracil: [(27-29); (30-40)]
thymine: [(31-33); (34-45)]
adenine: [(33-35); (36-47)]
guanine: [(38-39); (40-52)]

Here, the numbers refer to the orbital indices of the reference wave function. The intervals include the orbitals with orbital energies within the range: $[(\varepsilon_{\text{HOMO}} - \lambda_{\text{max}}); (\varepsilon_{\text{LUMO}} + \lambda_{\text{max}})]$, with $\varepsilon_{\text{HOMO}}$ and $\varepsilon_{\text{LUMO}}$ being the orbital energy of the highest occupied molecular orbital (HOMO) and lowest unoccupied molecular orbital (LUMO), respectively, and $\lambda_{\text{max}} = 0.1$ a.u. being the maximum applied interaction strength.

S5 Results

In the following, we present the results not provided in the manuscript. The results are grouped by molecule.

S5.1 Imidazole

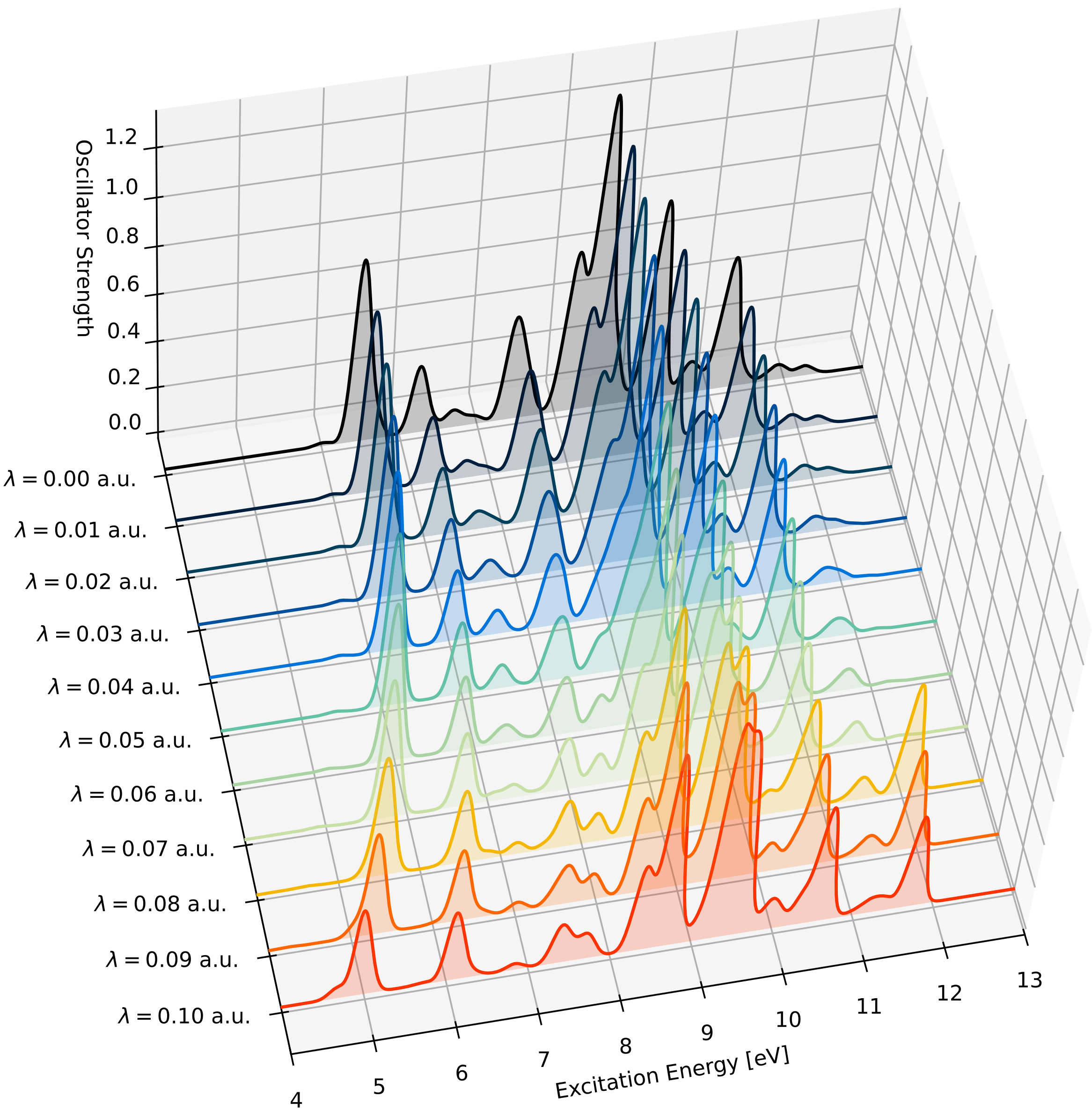

Figure S1: 3D representation of the valence absorption spectrum of imidazole. Each spectrum is constructed from 50 excitations by broadening with Gaussian functions with a standard deviation of 0.1 eV.

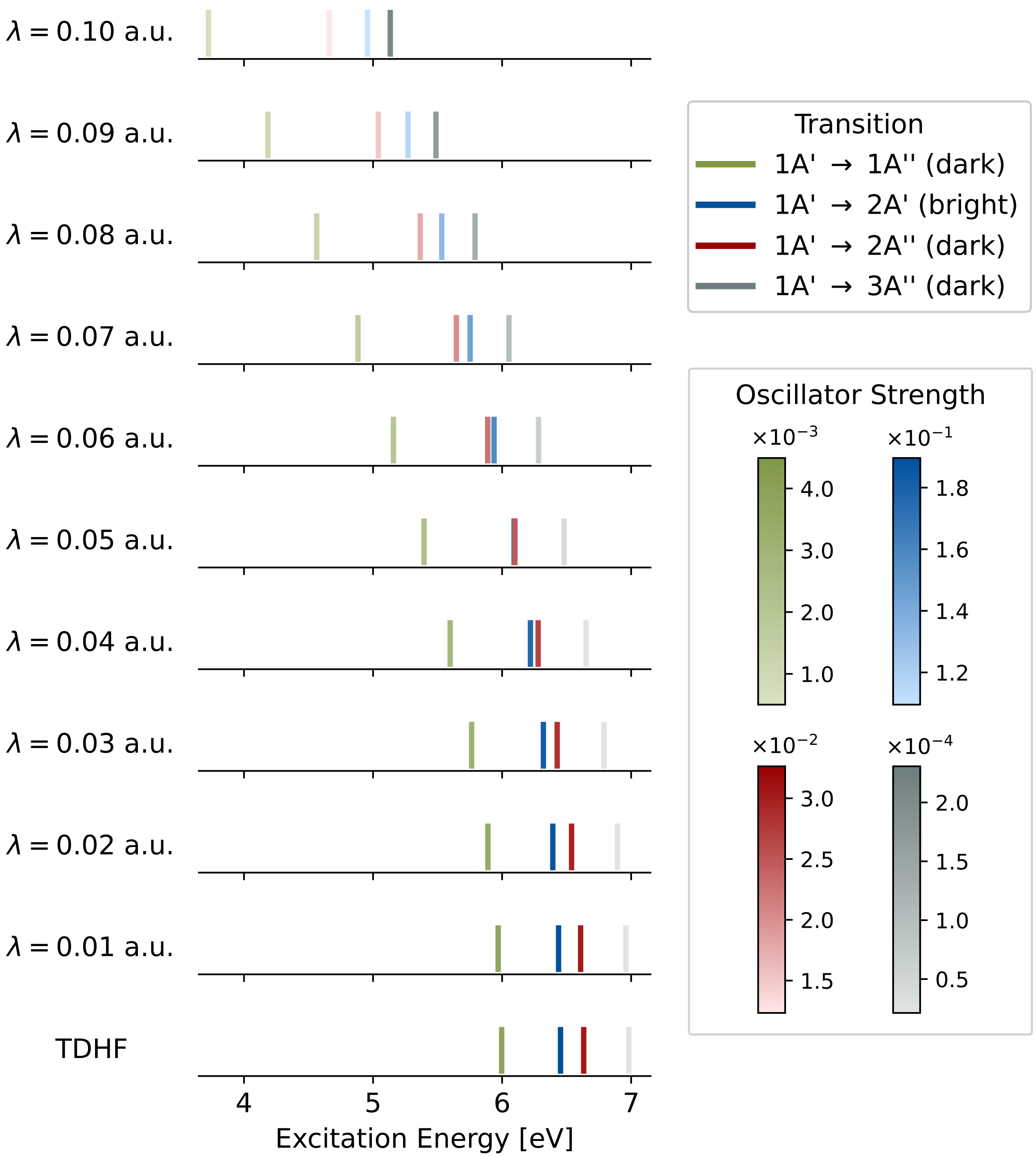

Figure S2: Stick spectrum representing the four lowest transitions in imidazole computed with different interaction strengths. The colors indicate the transitions, and the shades of each color represent the oscillator strengths.

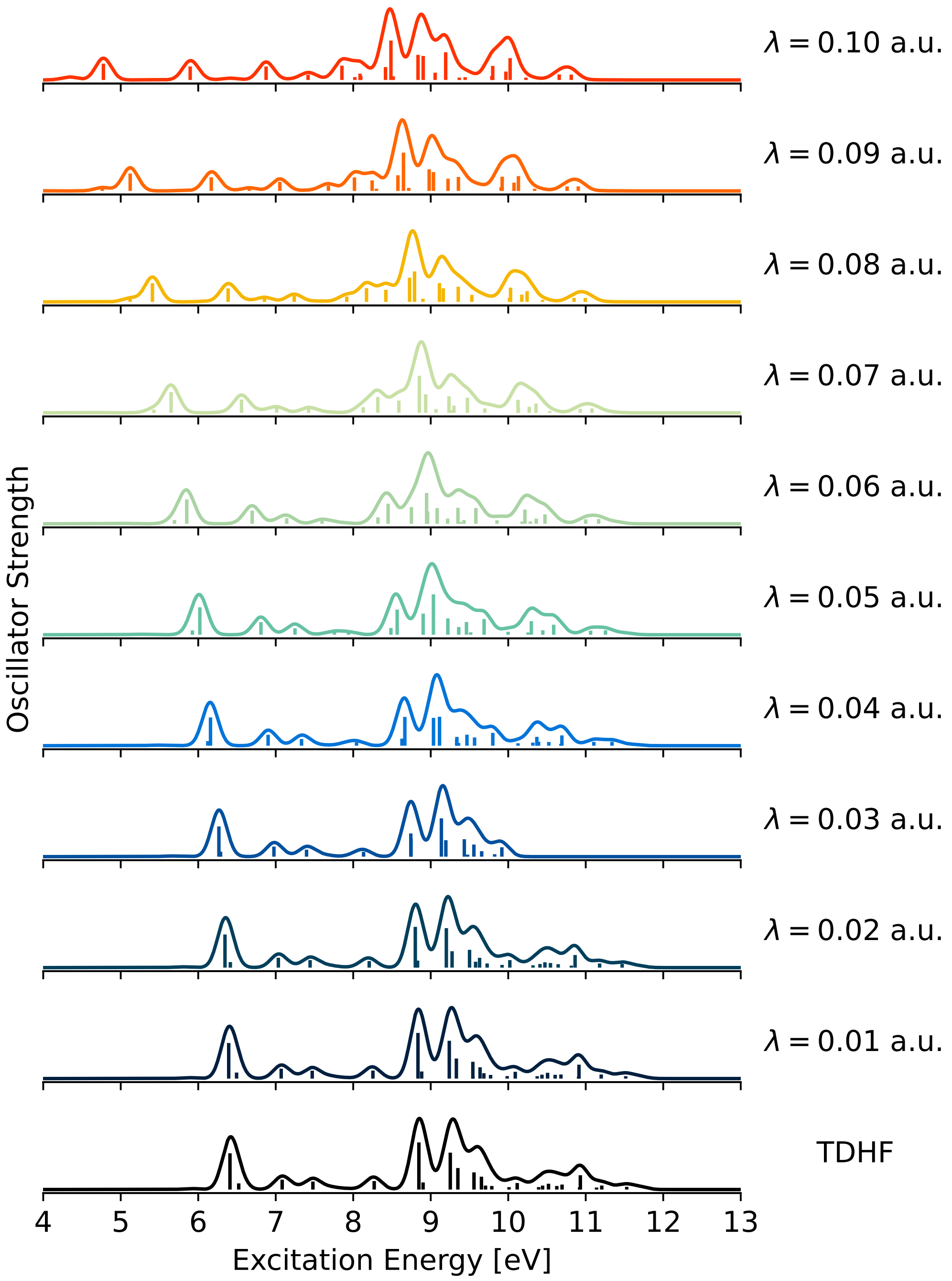

Figure S3: Valence absorption spectrum of imidazole computed with aug-cc-pVTZ for different interaction strengths. Each spectrum is constructed from 50 excitations by broadening with Gaussian functions with a standard deviation of 0.1 eV. Only sticks corresponding to $f_{\mathrm{osc}} \geq 0.01$ are visualized.

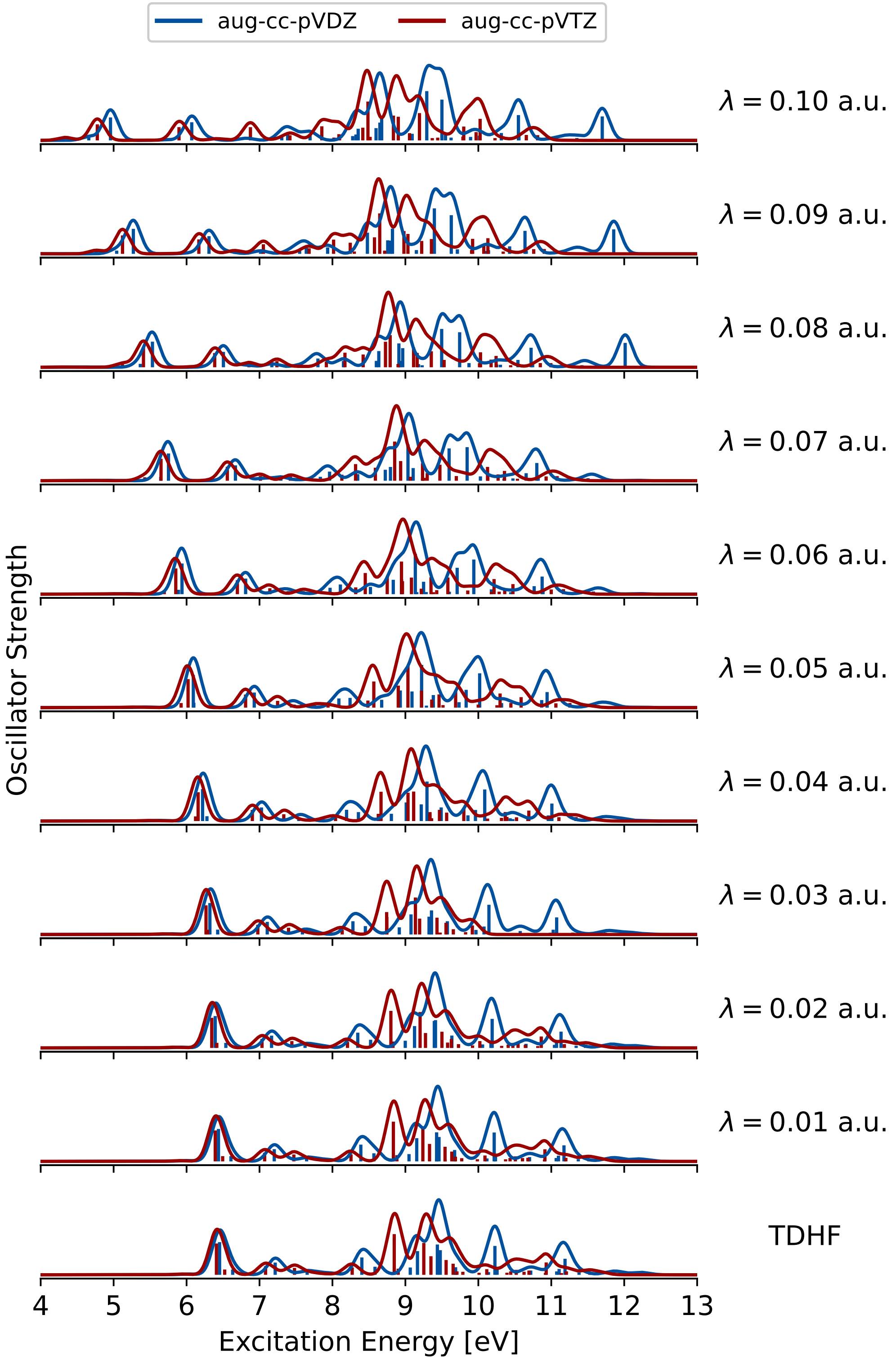

Figure S4: Comparison of valence absorption spectrum of imidazole computed with aug-cc-pVDZ and aug-cc-pVTZ for different interaction strengths.

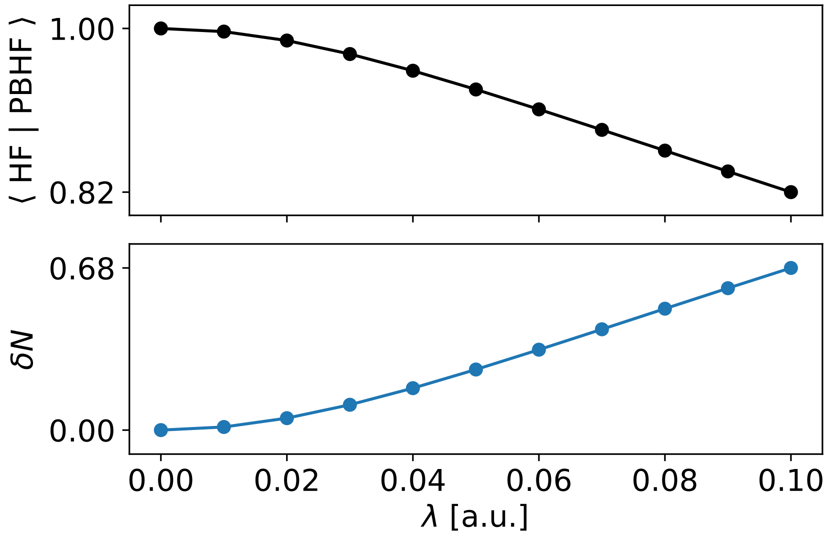

Figure S5: PBHF wave function overlap (top panel) and fractional charging (bottom panel) of imidazole computed with aug-cc-pVTZ as a function of environmental interaction strength.

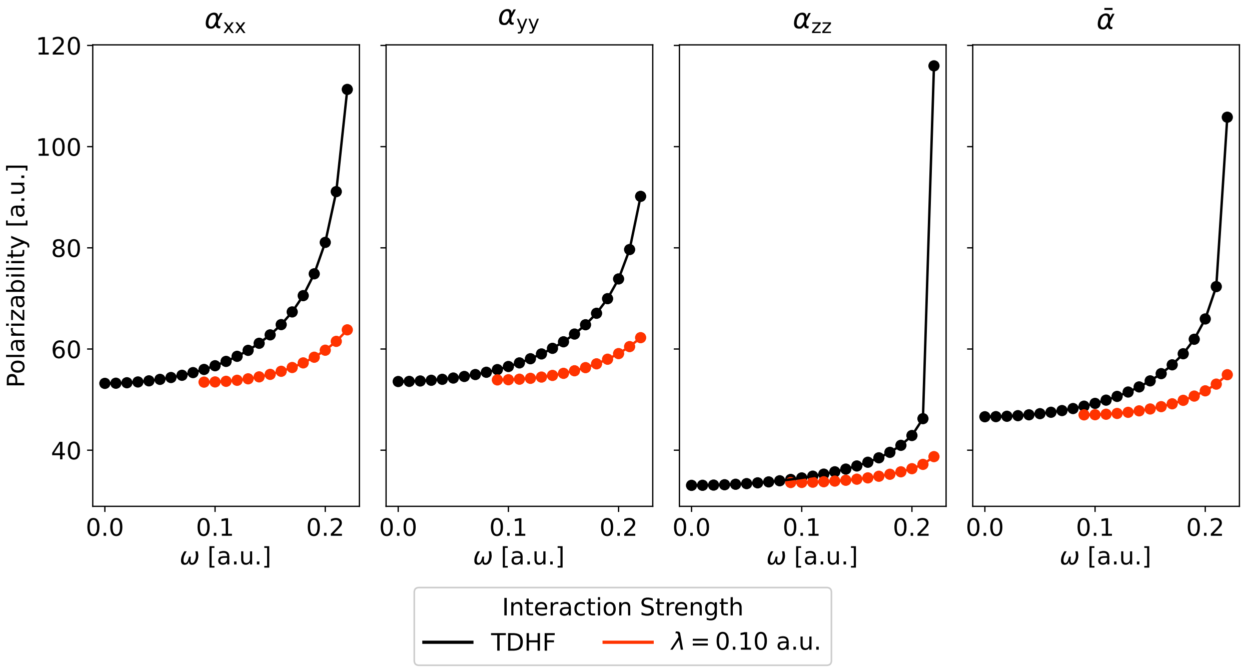

Figure S6: Shifted dispersion curve of the frequency-dependent electric dipole polarizability for imidazole.

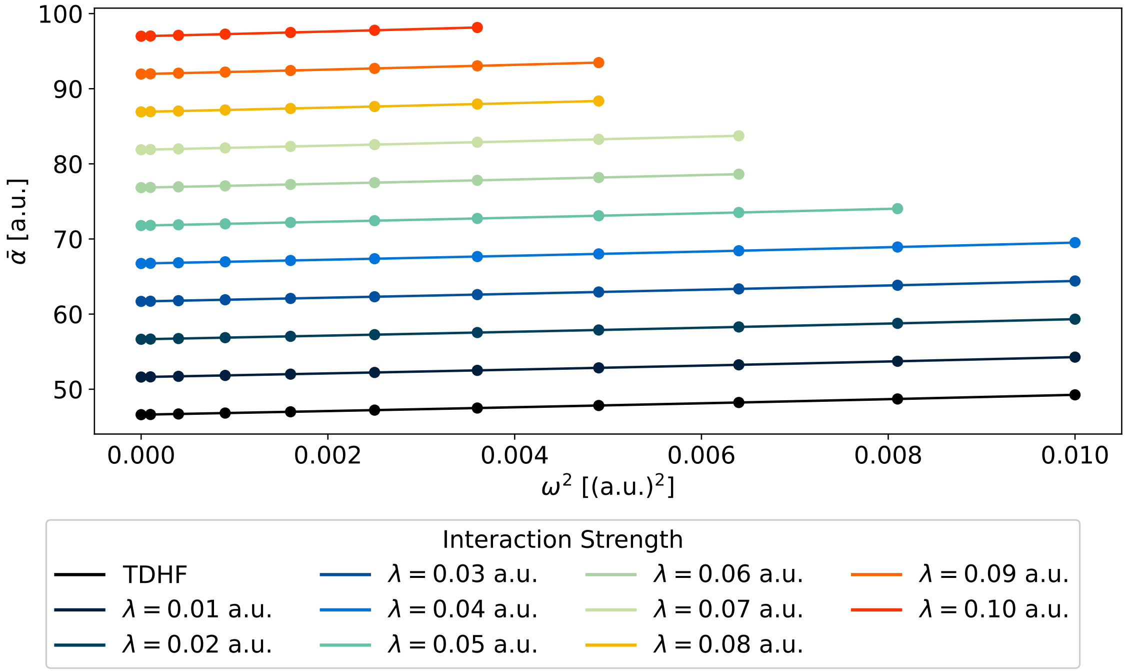

Figure S7: Linearized plot of the frequency-dependent electric dipole polarizability of imidazole computed with different interaction strengths. All curves computed for the electronically open molecule have been offset relative to the TDHF-computed curve.

Table S1: Estimated Cauchy moments for imidazole computed with aug-cc-pVDZ and aug-cc-pVTZ.

λ [a.u.]	$S(-2)$ [a.u.]		$S(-4)$ [a.u.]	
	aug-cc-pVDZ	aug-cc-pVTZ	aug-cc-pVDZ	aug-cc-pVTZ
0.00	46.59	47.11	261.64	270.03
0.01	46.60	47.12	262.49	270.87
0.02	46.62	47.15	265.01	273.32
0.03	46.66	47.20	269.11	277.25
0.04	46.71	47.25	274.73	282.57
0.05	46.76	47.31	275.62	282.84
0.06	46.82	47.37	278.15	284.69
0.07	46.86	47.43	287.91	293.82
0.08	46.91	47.49	294.03	299.13
0.09	46.94	47.54	308.97	313.39
0.10	46.98	47.60	322.04	325.85

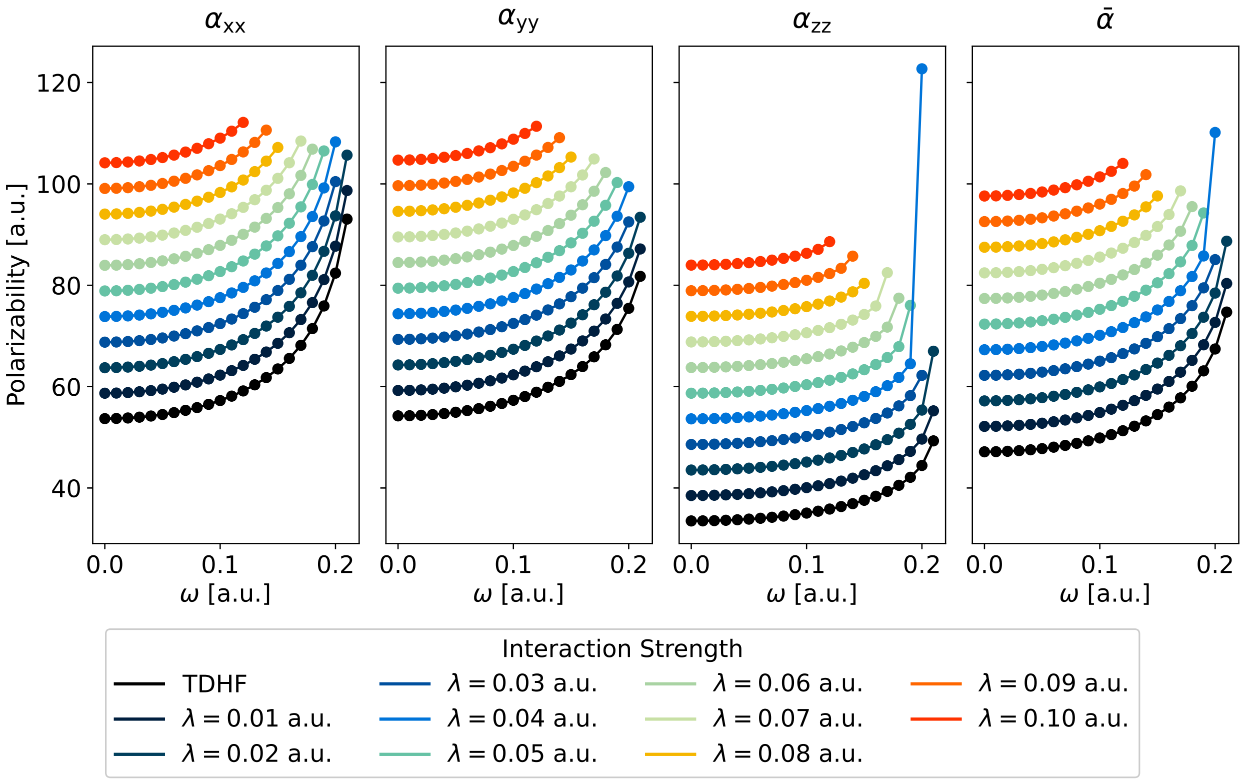

Figure S8: Dispersion curve of the frequency-dependent electric dipole polarizability of imidazole in the first resonance region computed with aug-cc-pVTZ. Each dispersion curve is vertically offset by 5 a.u. relative to the previous one for comparison purposes.

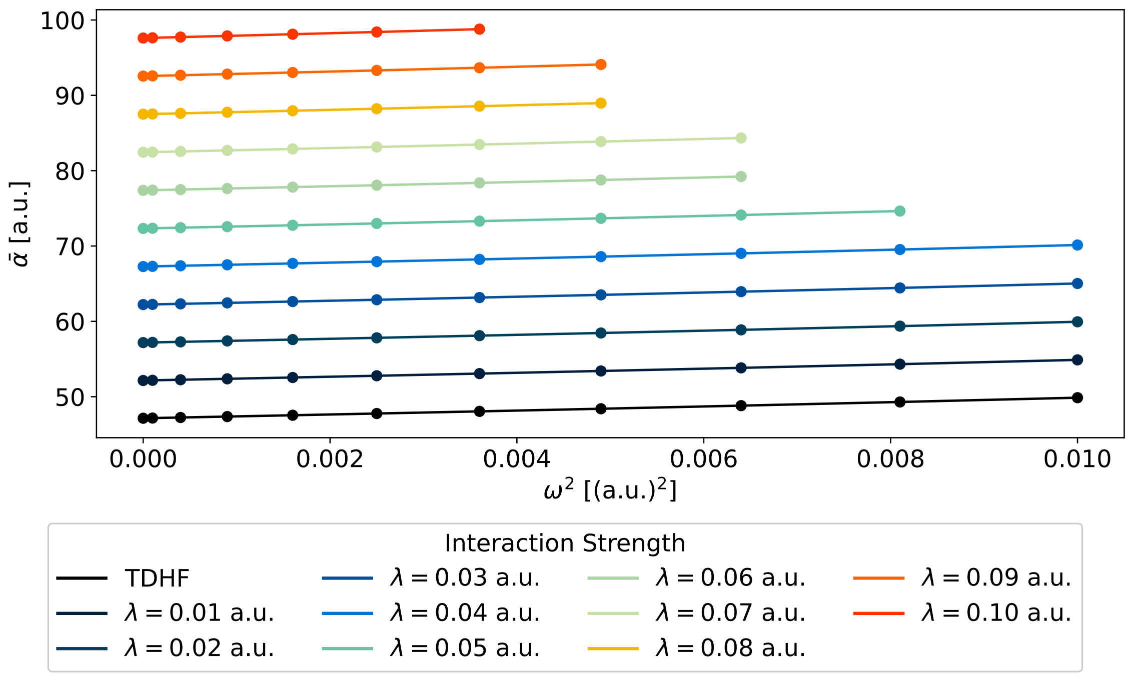

Figure S9: Linearized plot of the frequency-dependent electric dipole polarizability of imidazole computed with aug-cc-pVTZ for different interaction strengths. All curves computed for the electronically open molecule have been offset relative to the TDHF-computed curve.

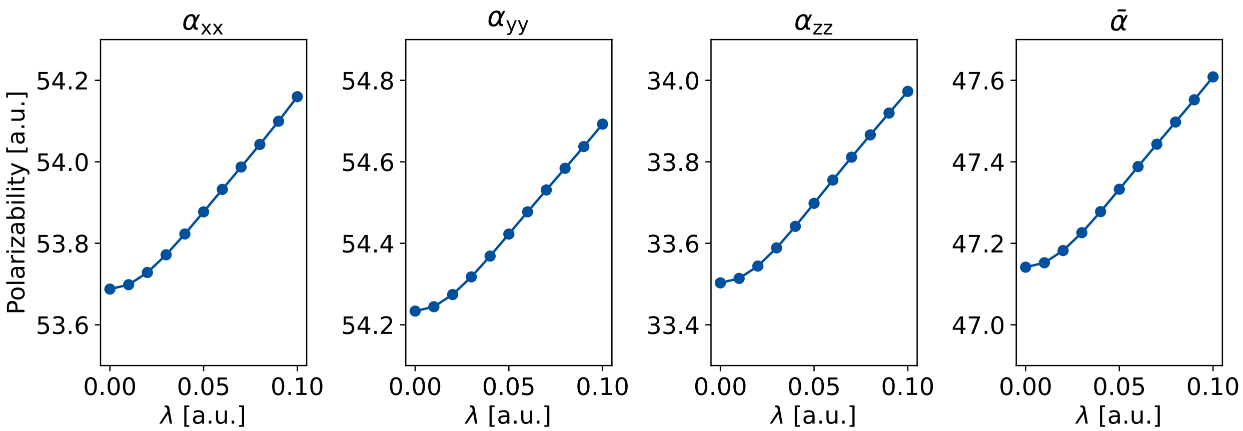

Figure S10: Static electric dipole polarizability of imidazole computed with aug-cc-pVTZ as a function of the interaction strength.

S5.2 Pyrazine

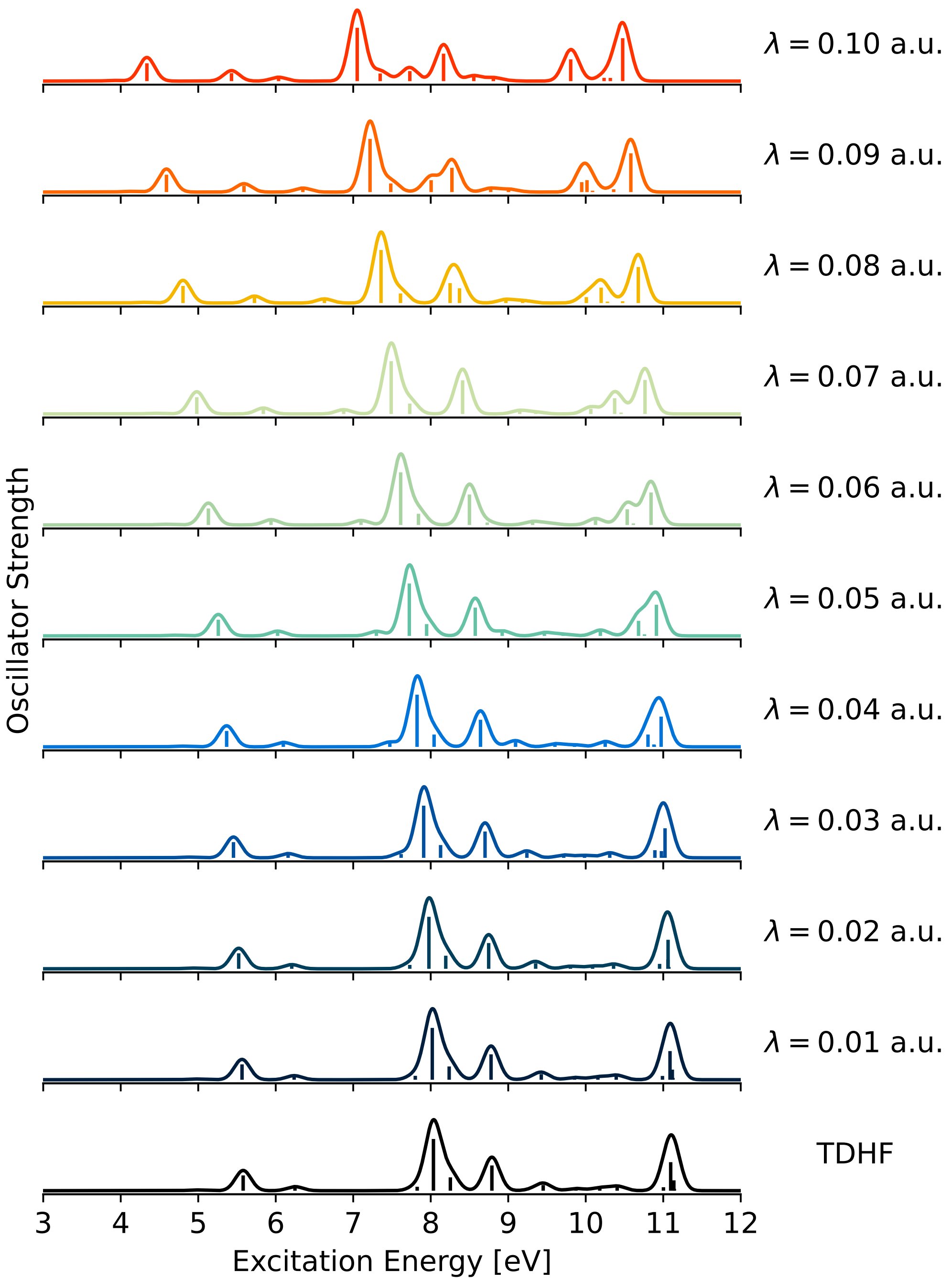

Figure S11: Valence absorption spectrum of pyrazine computed with different interaction strengths. Each spectrum is constructed from 50 excitations by broadening with Gaussian functions with a standard deviation of 0.1 eV. Only sticks corresponding to $f_{osc} \geq 0.01$ are visualized.

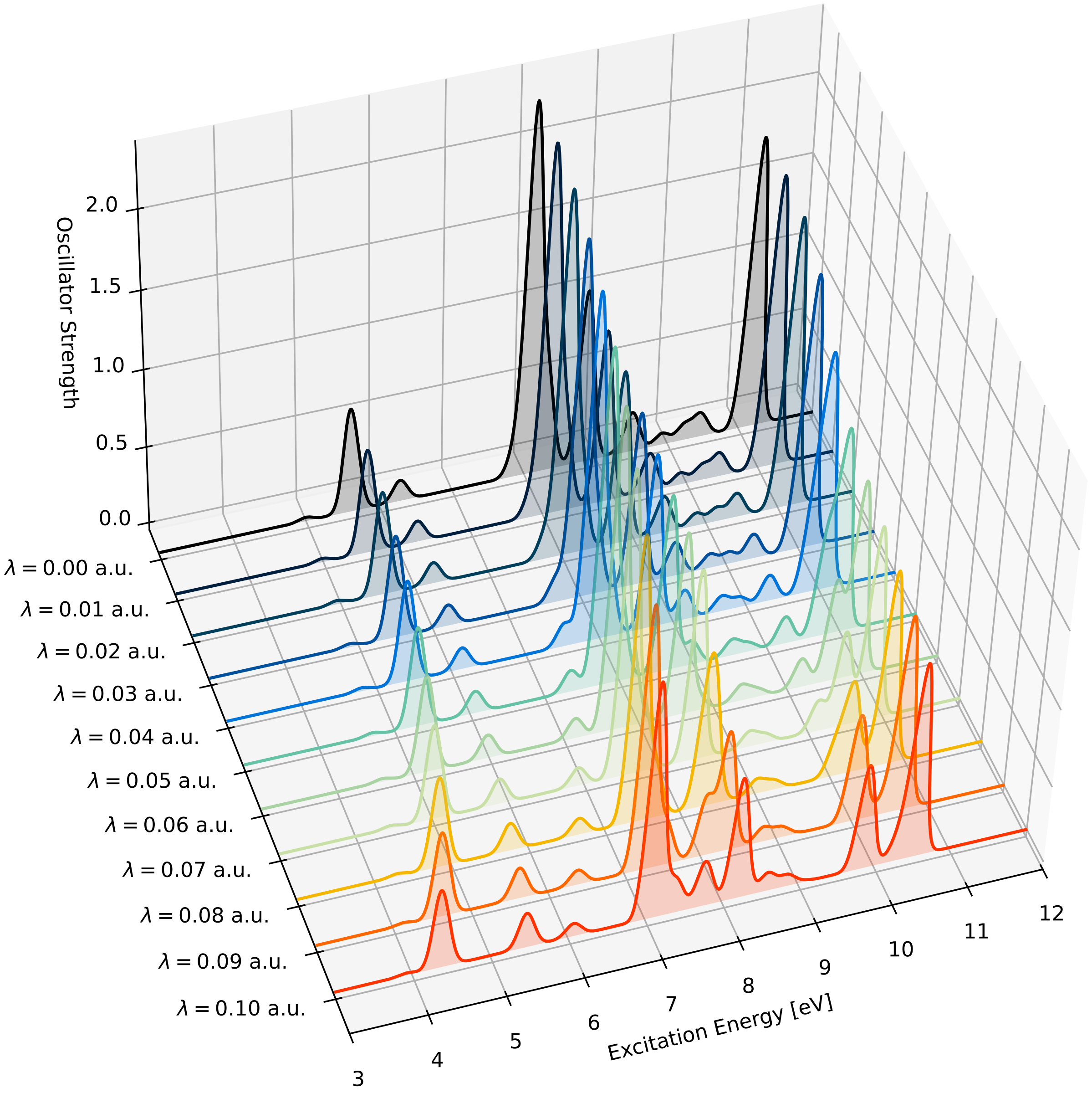

Figure S12: 3D representation of the valence absorption spectrum of pyrazine. Each spectrum is constructed from 50 excitations by broadening with Gaussian functions with a standard deviation of 0.1 eV.

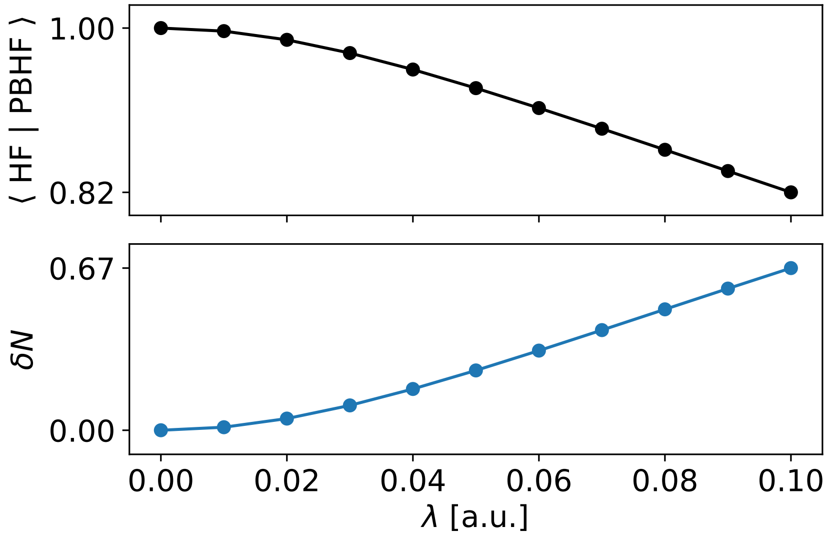

Figure S13: PBHF wave function overlap (top panel) and fractional charging (bottom panel) of pyrazine as a function of environmental interaction strength.

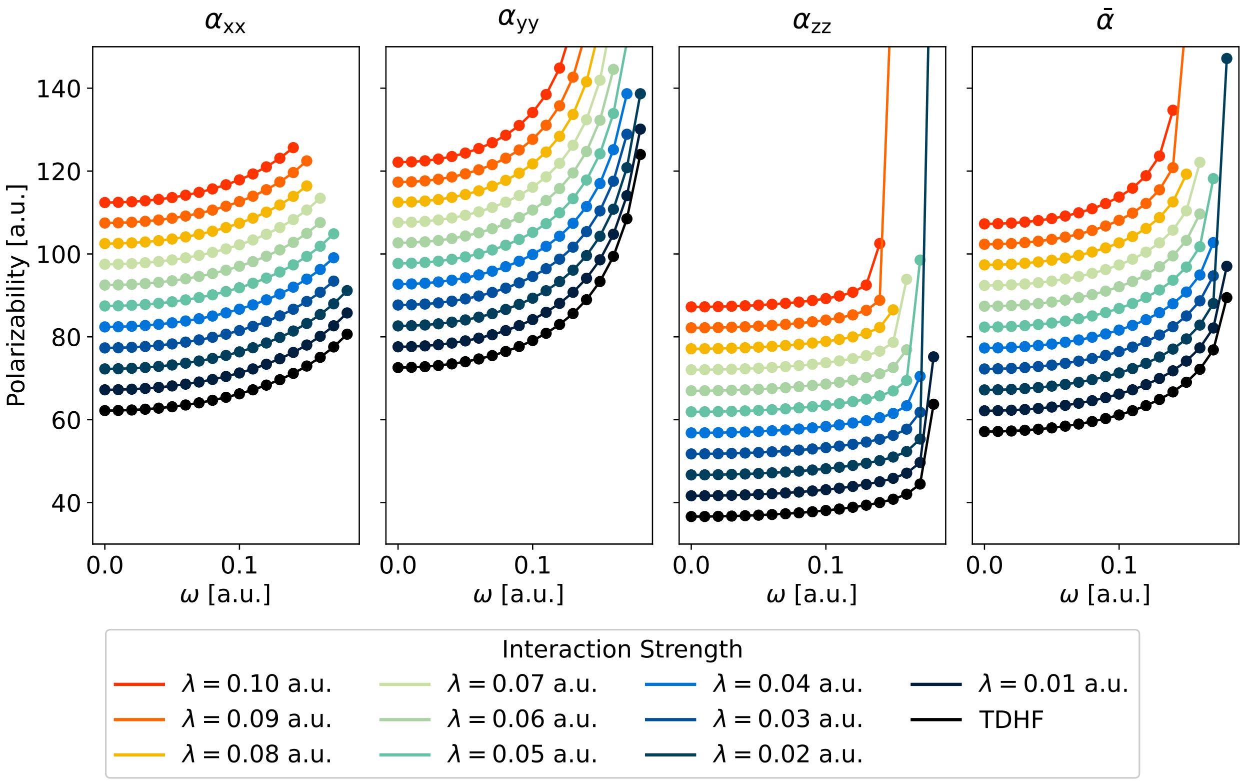

Figure S14: Dispersion curve of the frequency-dependent electric dipole polarizability of pyrazine in the first resonance region. Each dispersion curve is vertically offset by 5 a.u. relative to the previous one for comparison purposes.

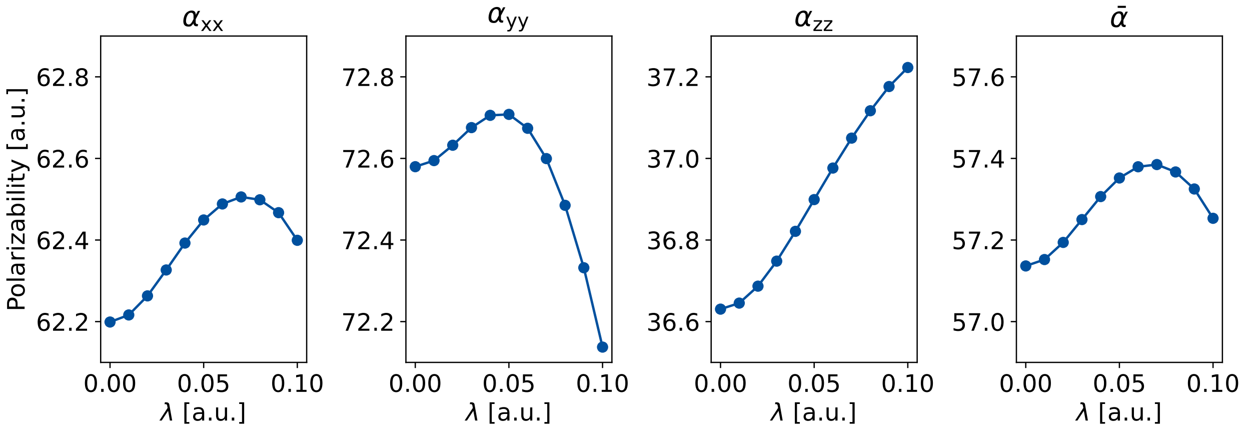

Figure S15: Static electric dipole polarizability of pyrazine as a function of the interaction strength.

S5.3 Cytosine

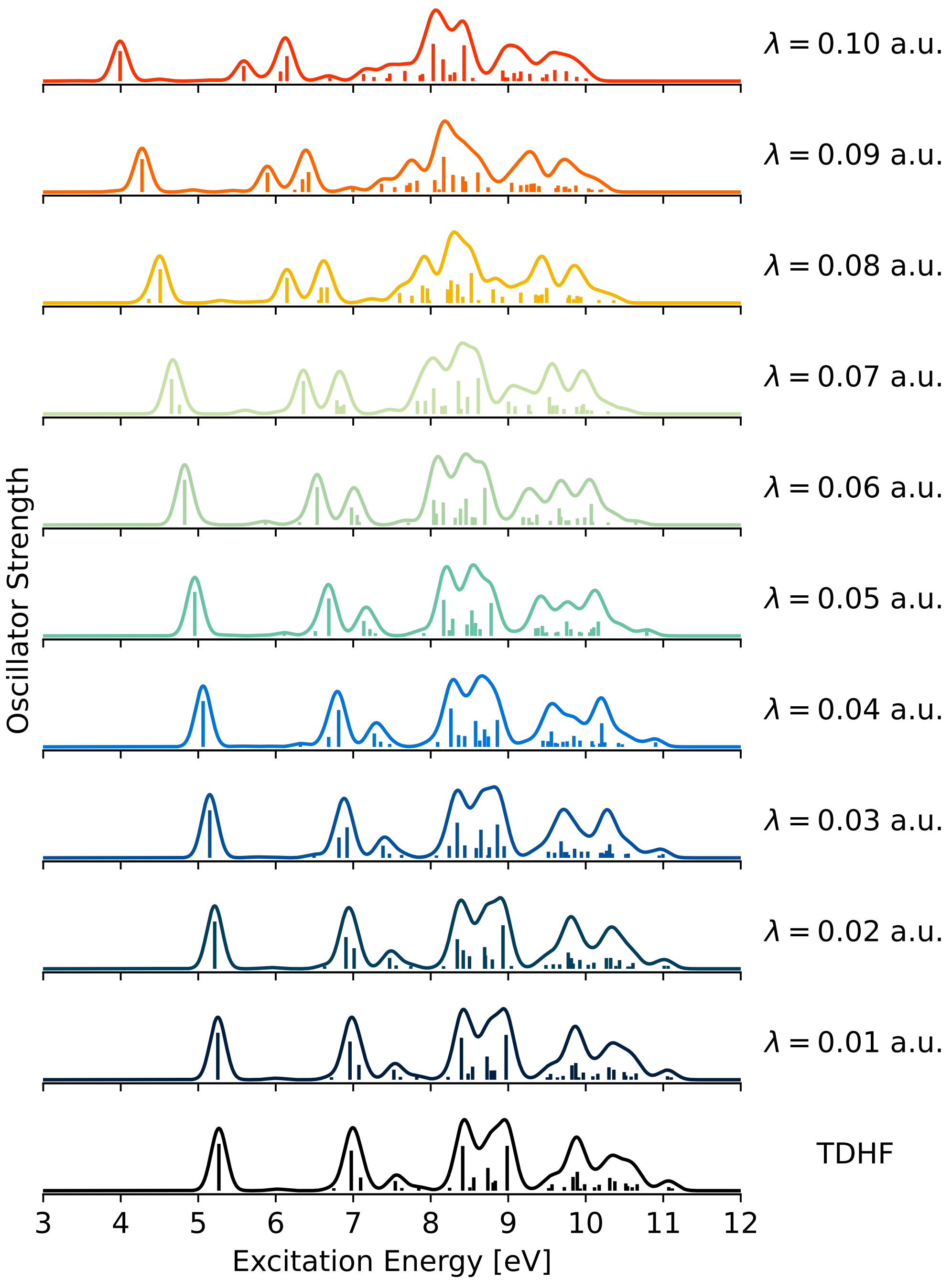

Figure S16: Valence absorption spectrum of cytosine computed with different interaction strengths. Each spectrum is constructed from 50 excitations by broadening with Gaussian functions with a standard deviation of 0.1 eV. Only sticks corresponding to $f_{\text{osc}} \geq 0.01$ are visualized.

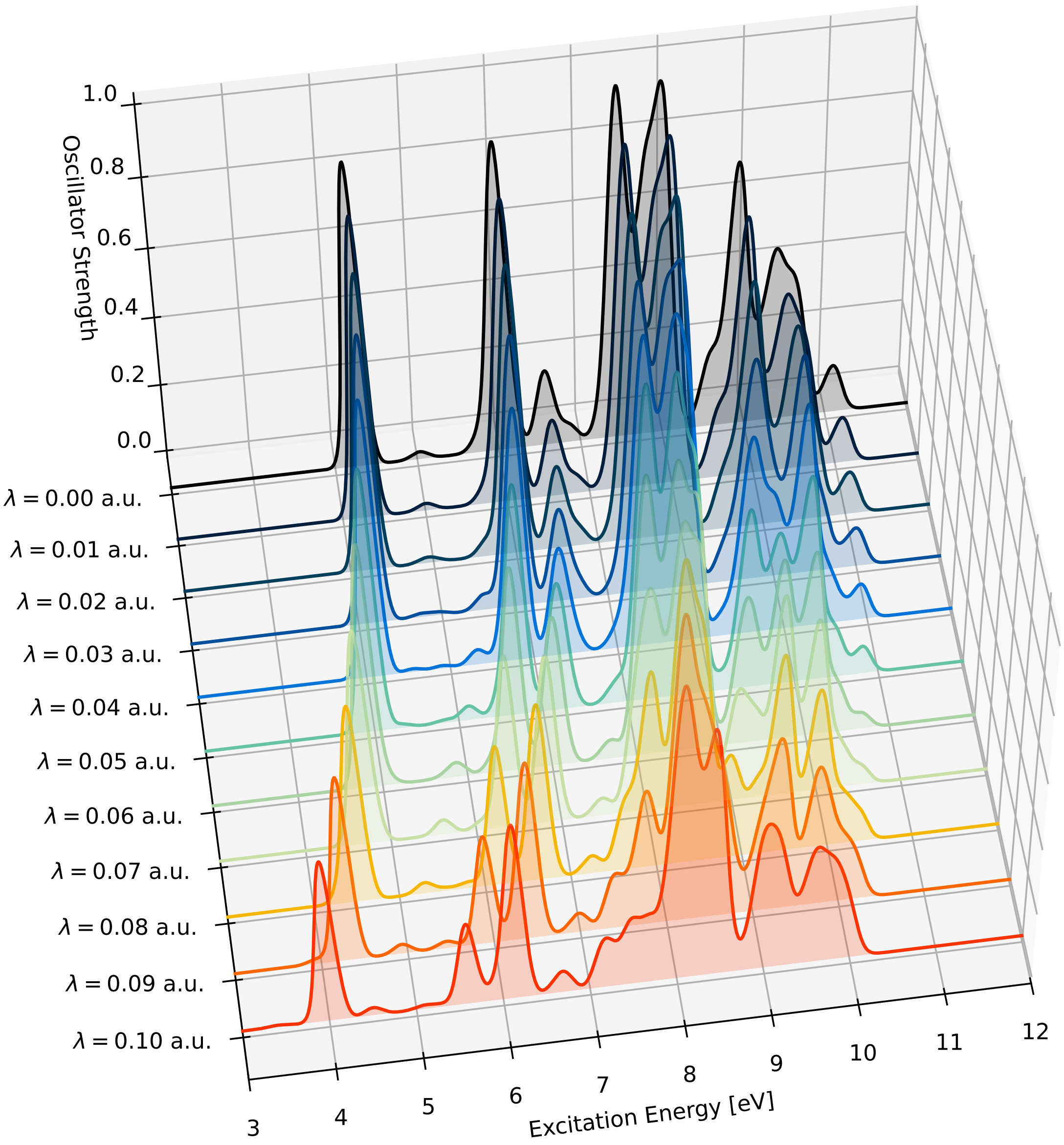

Figure S17: 3D representation of the valence absorption spectrum of cytosine. Each spectrum is constructed from 50 excitations by broadening with Gaussian functions with a standard deviation of 0.1 eV.

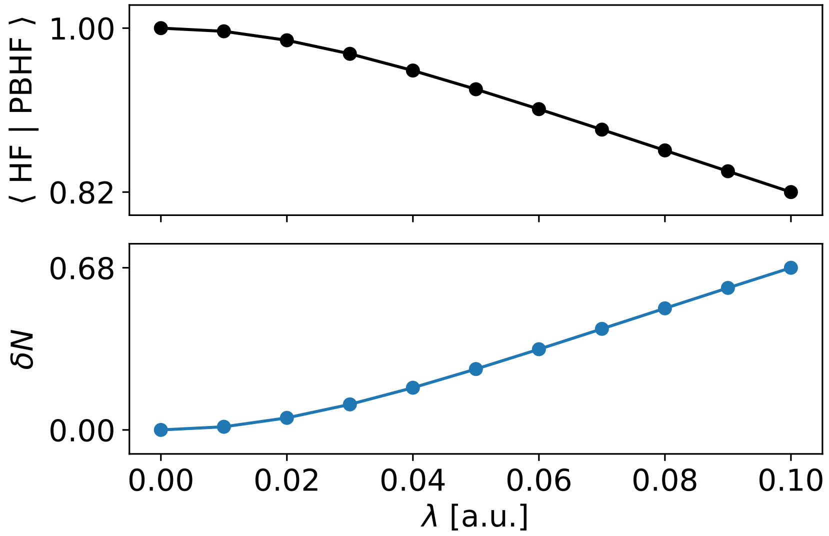

Figure S18: PBHF wave function overlap (top panel) and fractional charging (bottom panel) of cytosine as a function of environmental interaction strength.

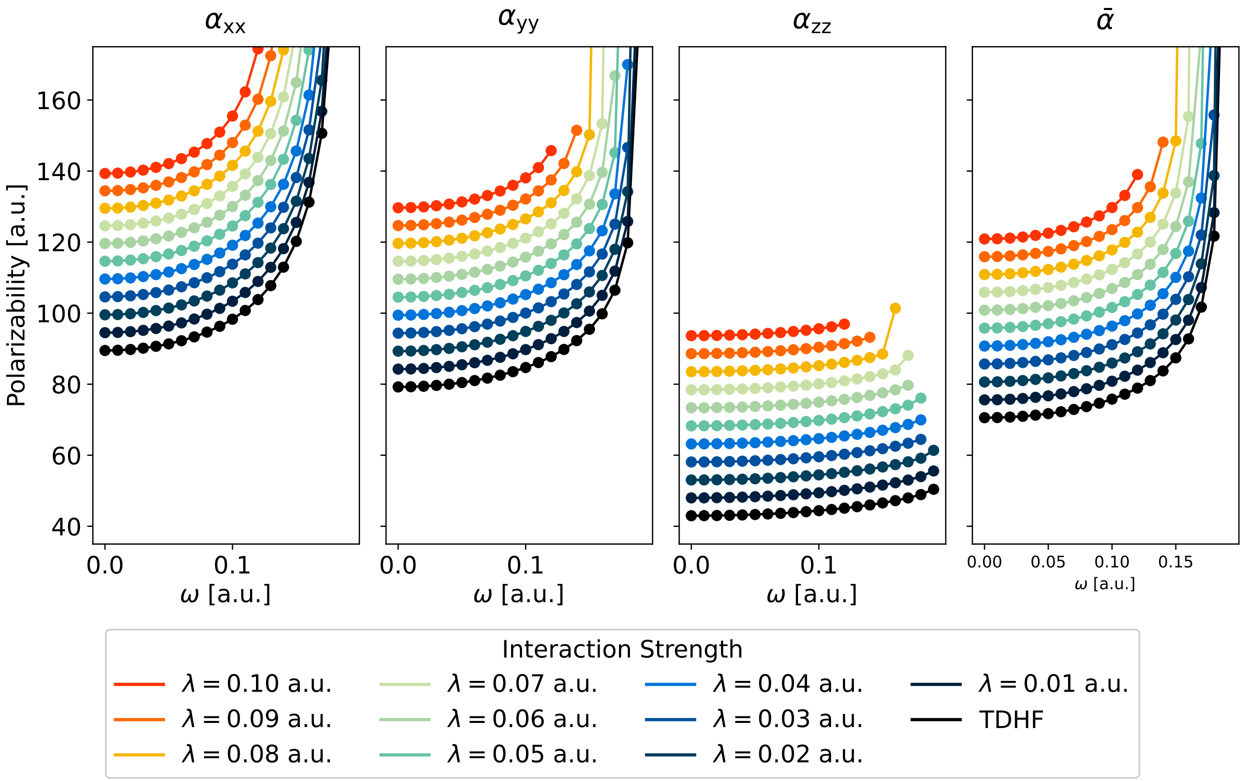

Figure S19: Dispersion curve of the frequency-dependent electric dipole polarizability of cytosine in the first resonance region. Each dispersion curve is vertically offset by 5 a.u. relative to the previous one for comparison purposes.

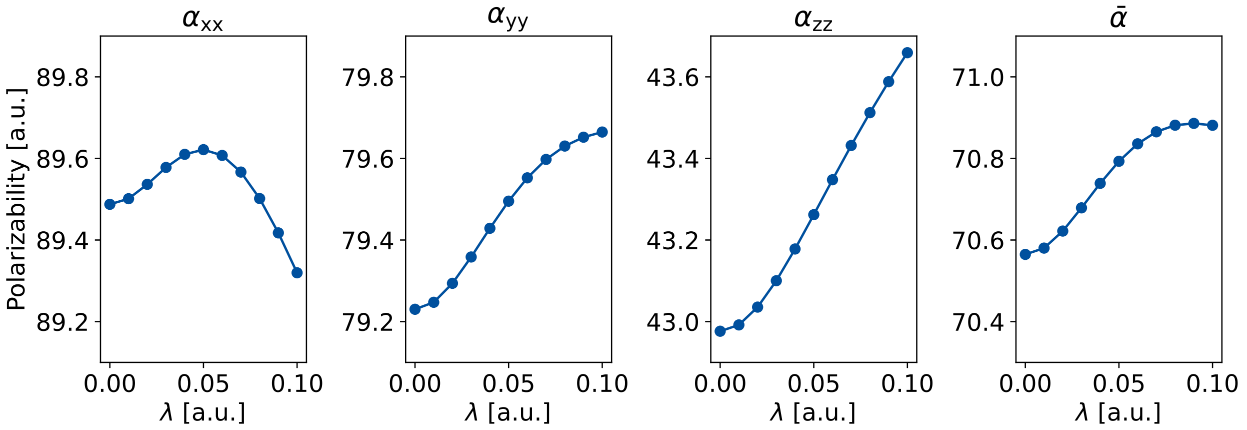

Figure S20: Static electric dipole polarizability of cytosine as a function of the interaction strength.

S5.4 Uracil

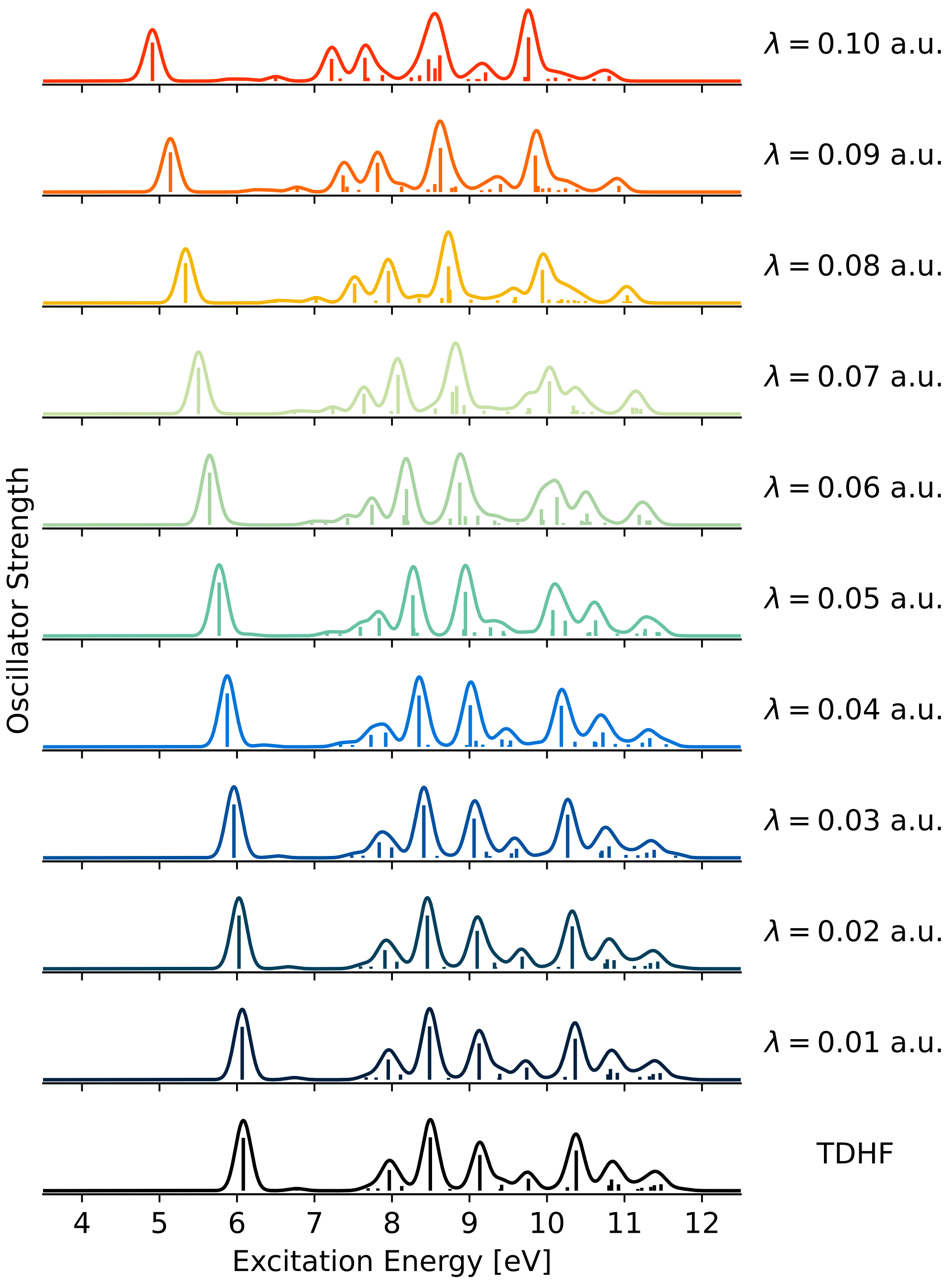

Figure S21: Valence absorption spectrum of uracil computed with different interaction strengths. Each spectrum is constructed from 50 excitations by broadening with Gaussian functions with a standard deviation of 0.1 eV. Only sticks corresponding to $f_{\text{osc}} \geq 0.01$ are visualized.

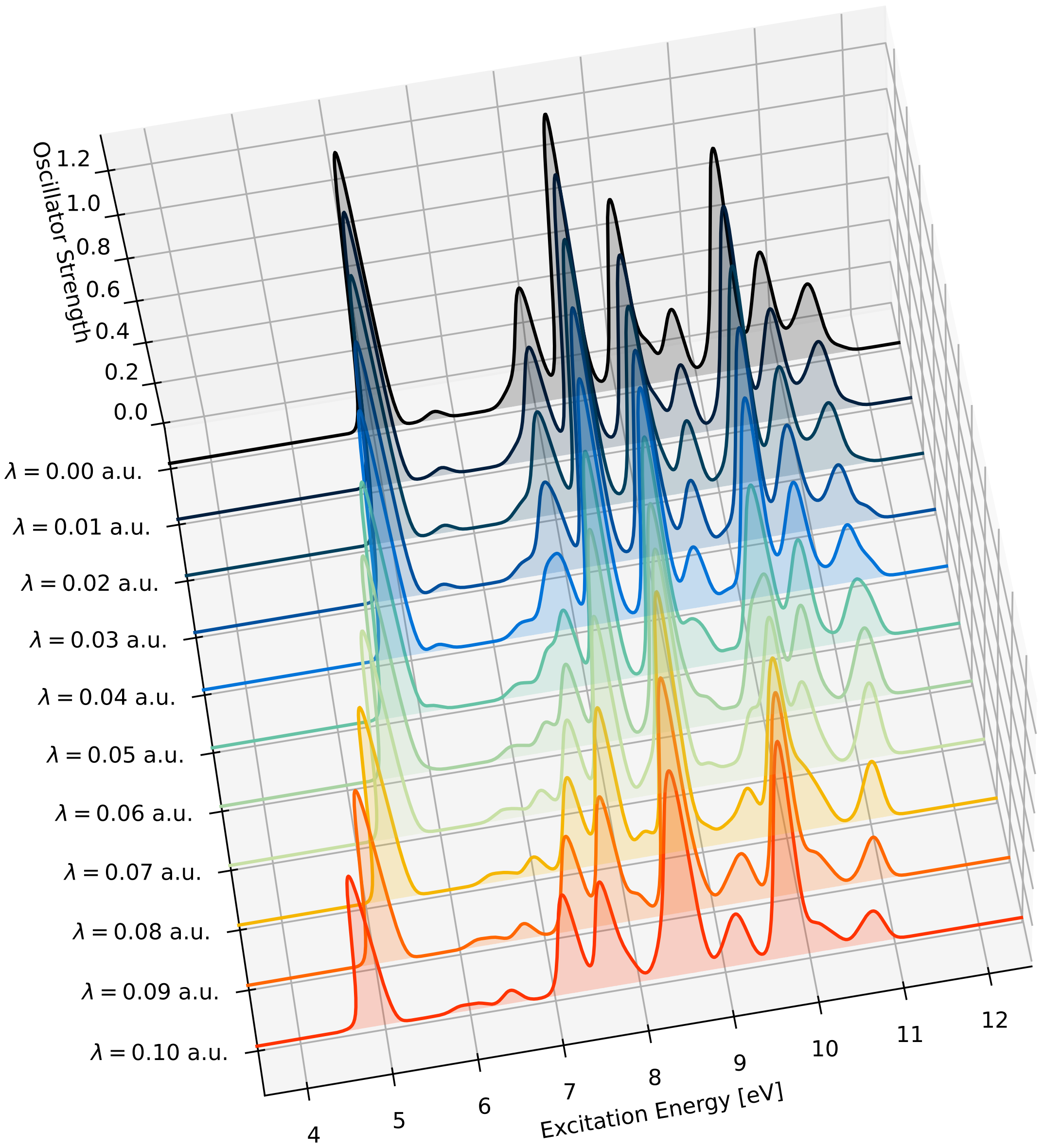

Figure S22: 3D representation of the valence absorption spectrum of uracil. Each spectrum is constructed from 50 excitations by broadening with Gaussian functions with a standard deviation of 0.1 eV.

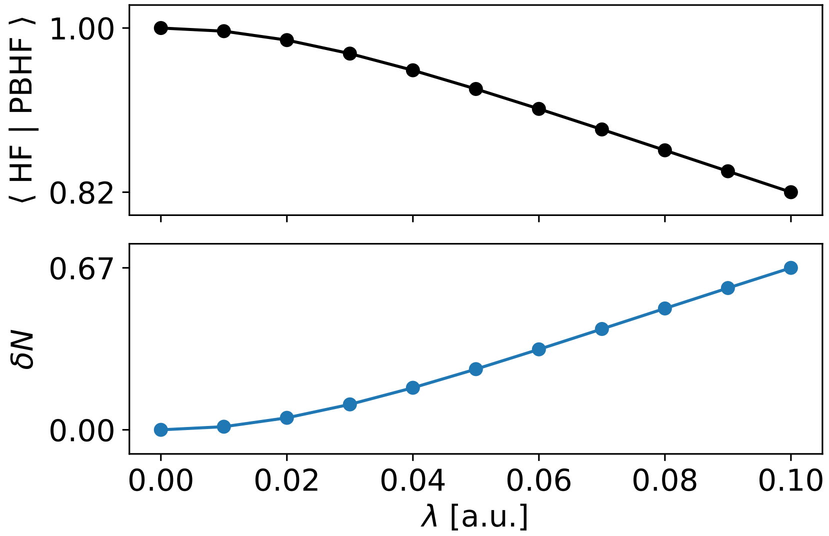

Figure S23: PBHF wave function overlap (top panel) and fractional charging (bottom panel) of uracil as a function of environmental interaction strength.

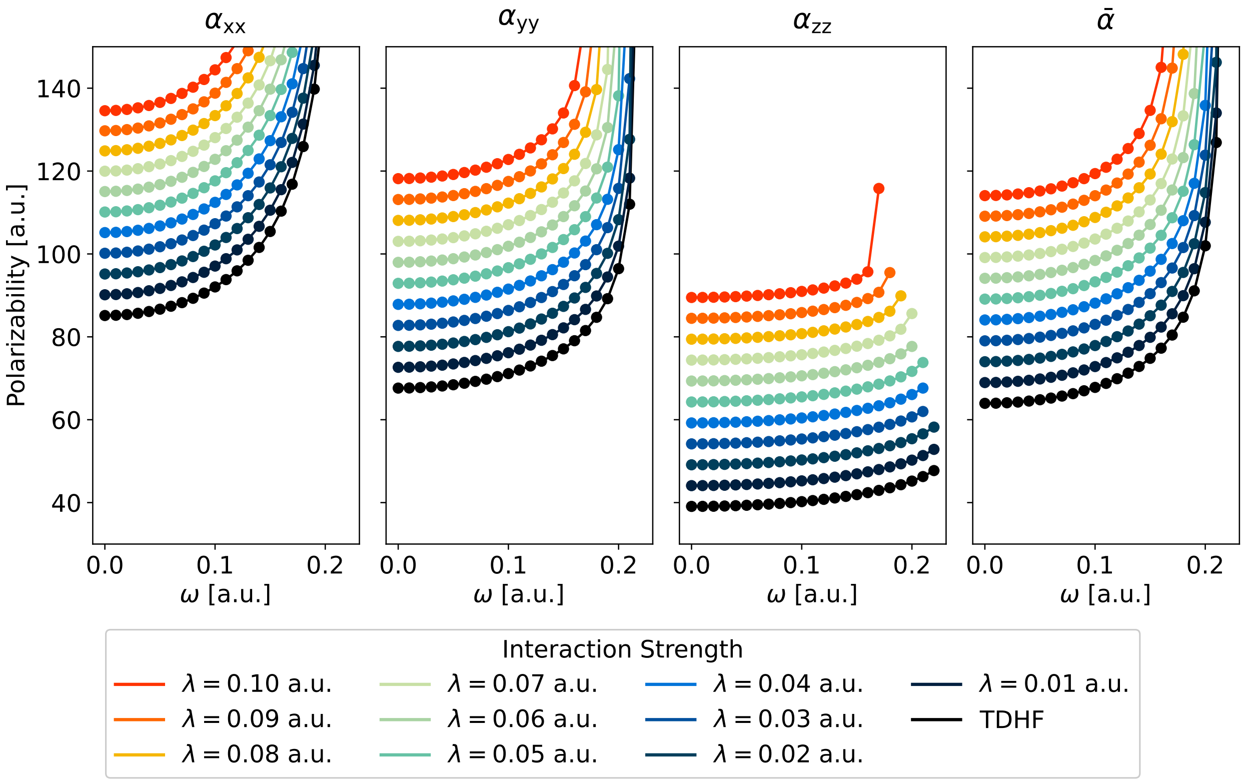

Figure S24: Dispersion curve of the frequency-dependent electric dipole polarizability of uracil in the first resonance region. Each dispersion curve is vertically offset by 5 a.u. relative to the previous one for comparison purposes.

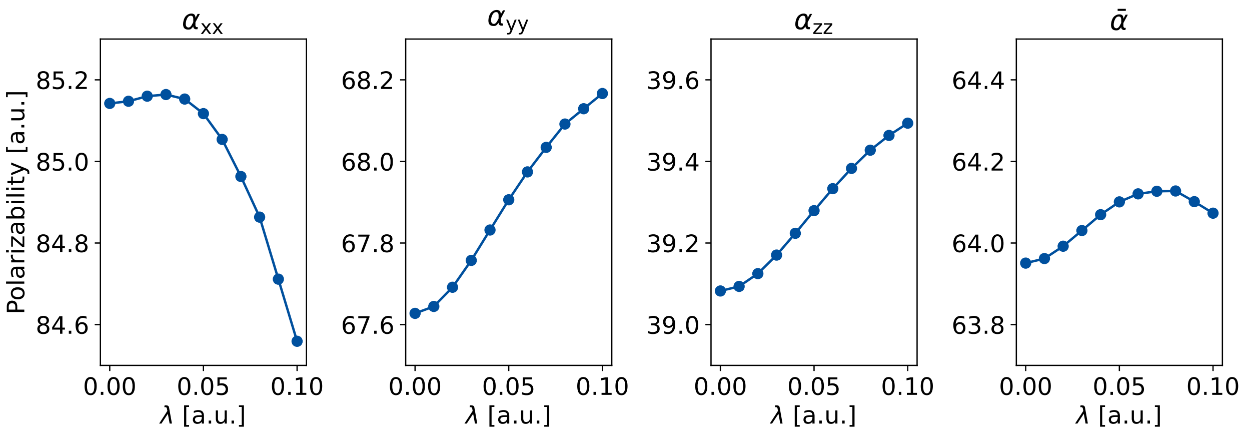

Figure S25: Static electric dipole polarizability of uracil as a function of the interaction strength.

S5.5 Thymine

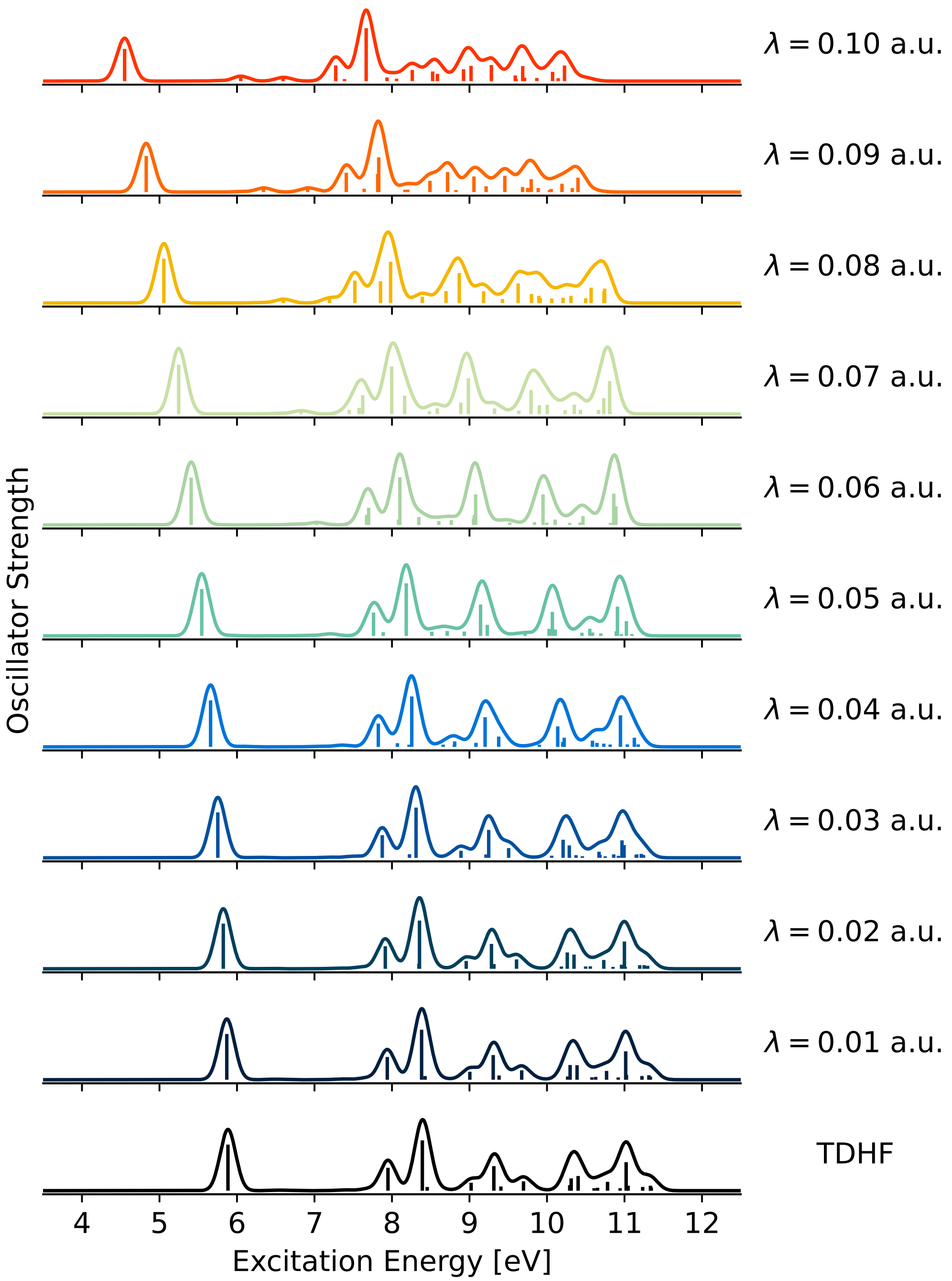

Figure S26: Valence absorption spectrum of thymine computed with different interaction strengths. Each spectrum is constructed from 50 excitations by broadening with Gaussian functions with a standard deviation of 0.1 eV. Only sticks corresponding to $f_{\mathrm{osc}} \geq 0.01$ are visualized.

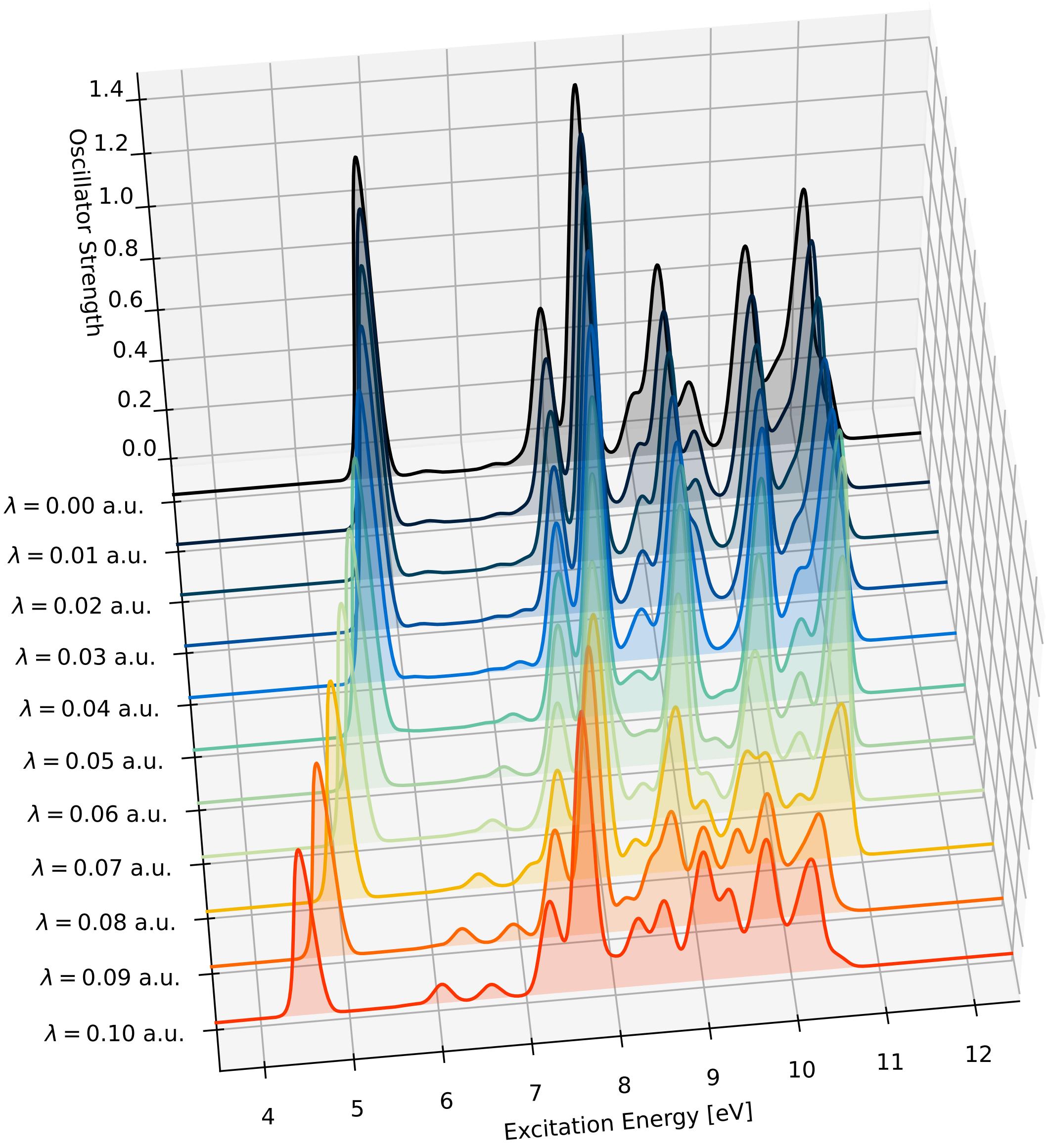

Figure S27: 3D representation of the valence absorption spectrum of thymine. Each spectrum is constructed from 50 excitations by broadening with Gaussian functions with a standard deviation of 0.1 eV.

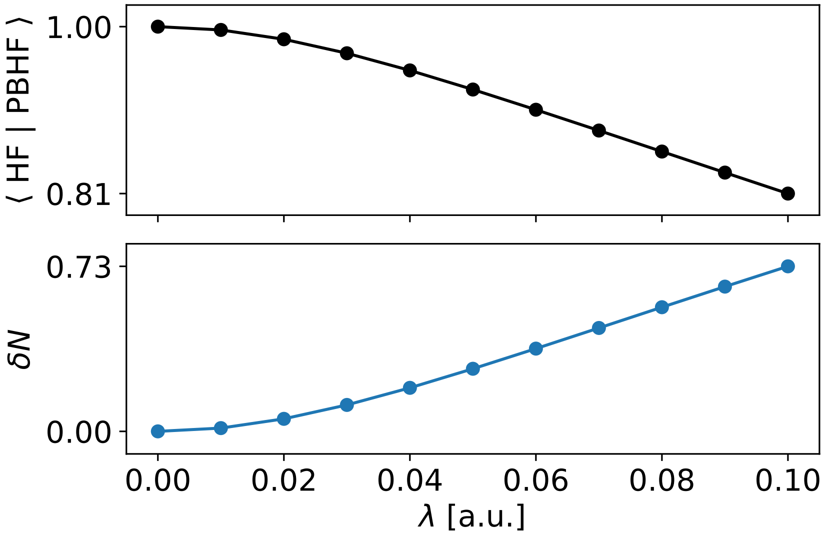

Figure S28: PBHF wave function overlap (top panel) and fractional charging (bottom panel) of thymine as a function of environmental interaction strength.

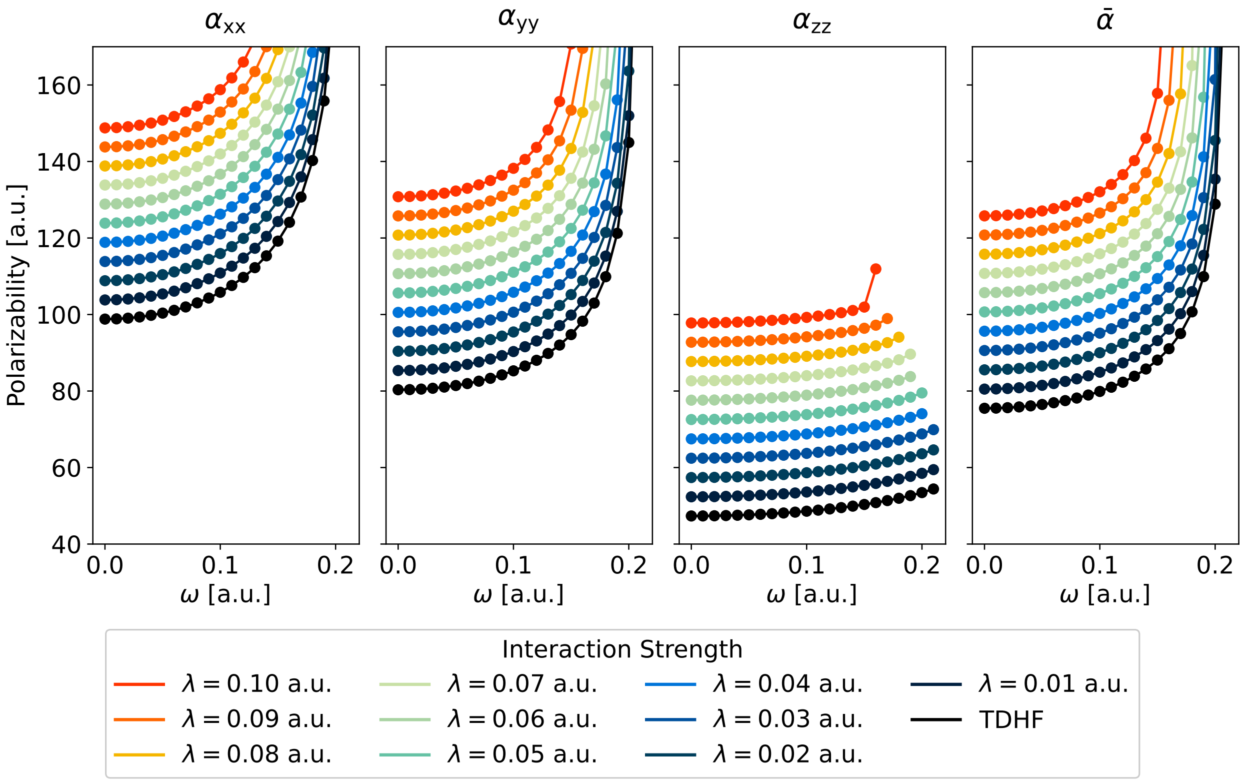

Figure S29: Dispersion curve of the frequency-dependent electric dipole polarizability of thymine in the first resonance region. Each dispersion curve is vertically offset by 5 a.u. relative to the previous one for comparison purposes.

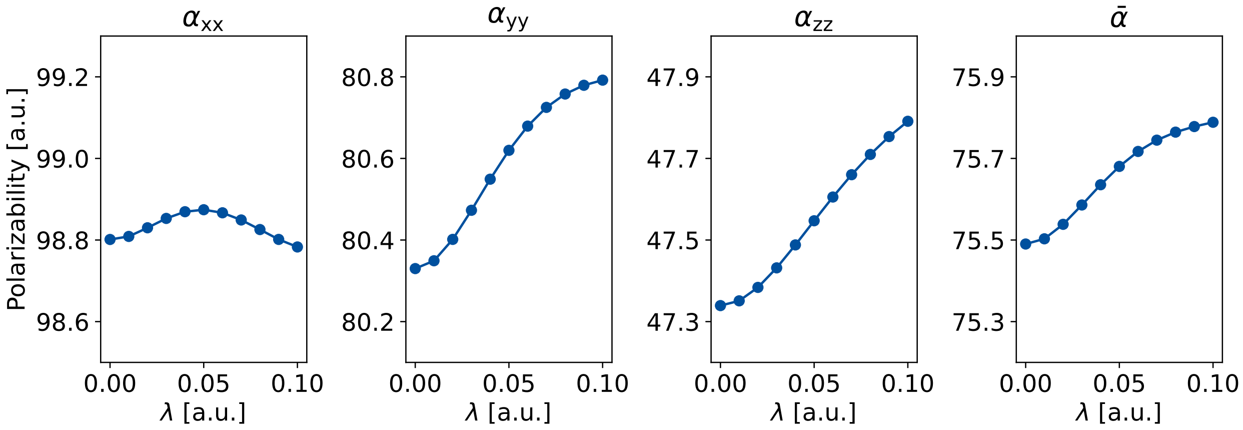

Figure S30: Static electric dipole polarizability of thymine as a function of the interaction strength.

S5.6 Adenine

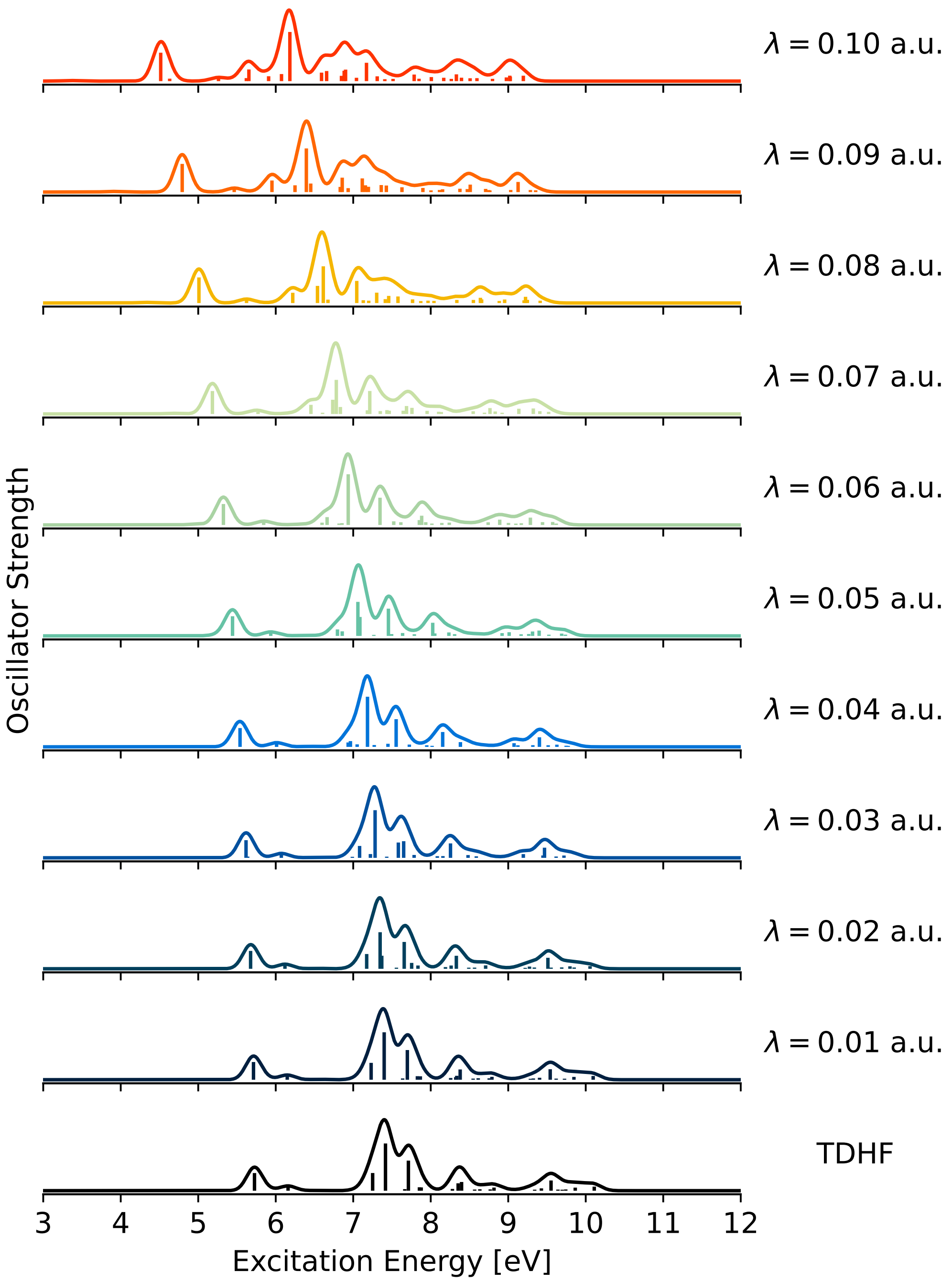

Figure S31: Valence absorption spectrum of adenine computed with different interaction strengths. Each spectrum is constructed from 50 excitations by broadening with Gaussian functions with a standard deviation of 0.1 eV. Only sticks corresponding to $f_{\text{osc}} \geq 0.01$ are visualized.

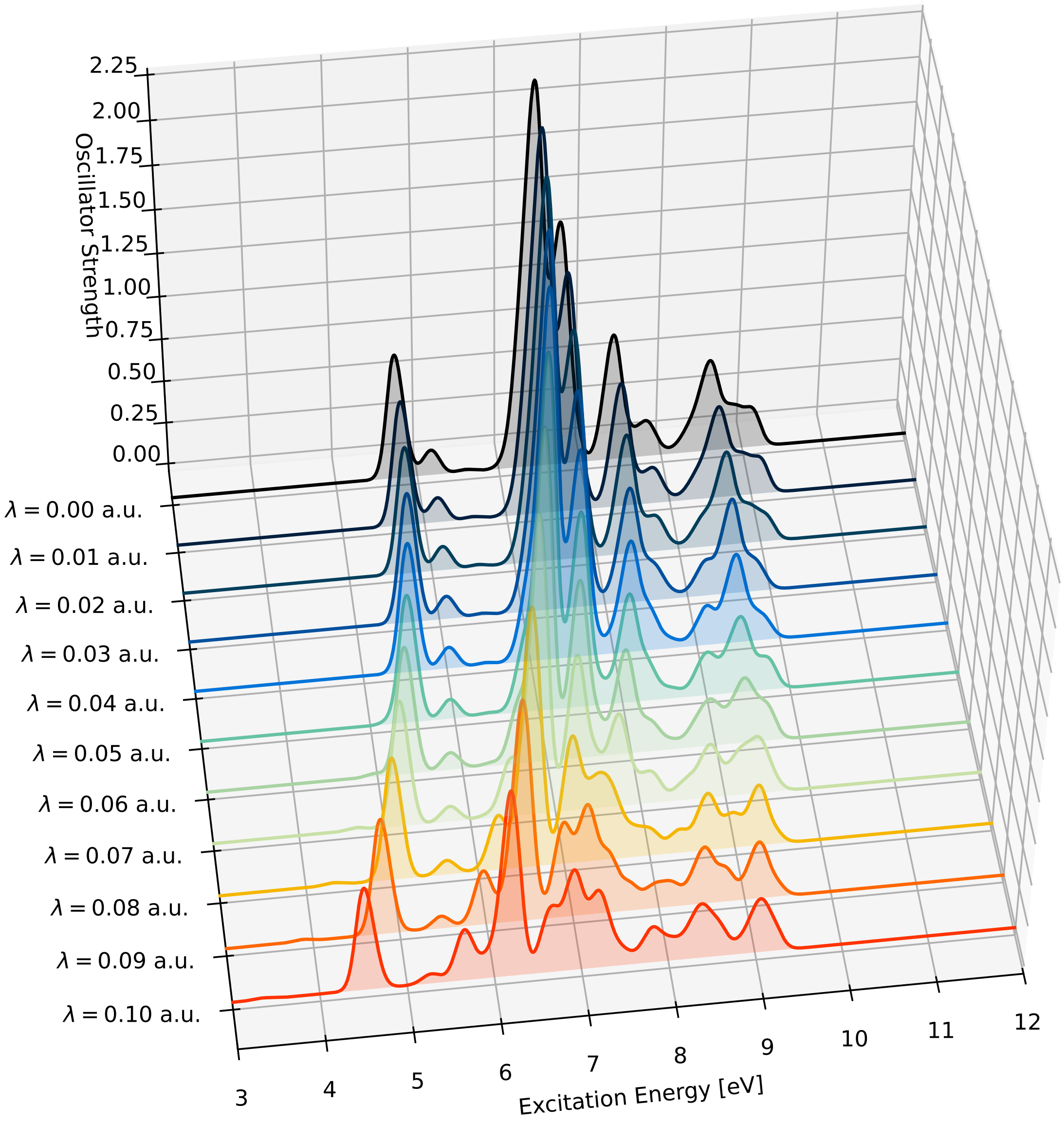

Figure S32: 3D representation of the valence absorption spectrum of adenine. Each spectrum is constructed from 50 excitations by broadening with Gaussian functions with a standard deviation of 0.1 eV.

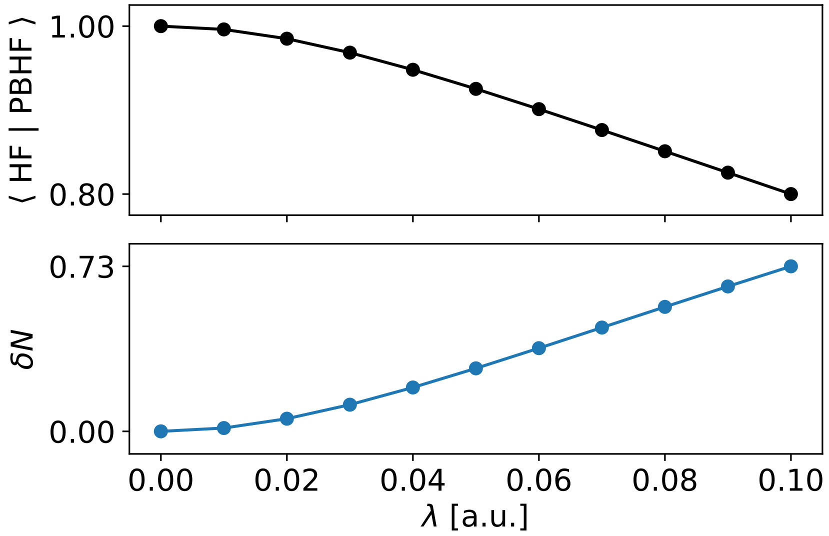

Figure S33: PBHF wave function overlap (top panel) and fractional charging (bottom panel) of adenine as a function of environmental interaction strength.

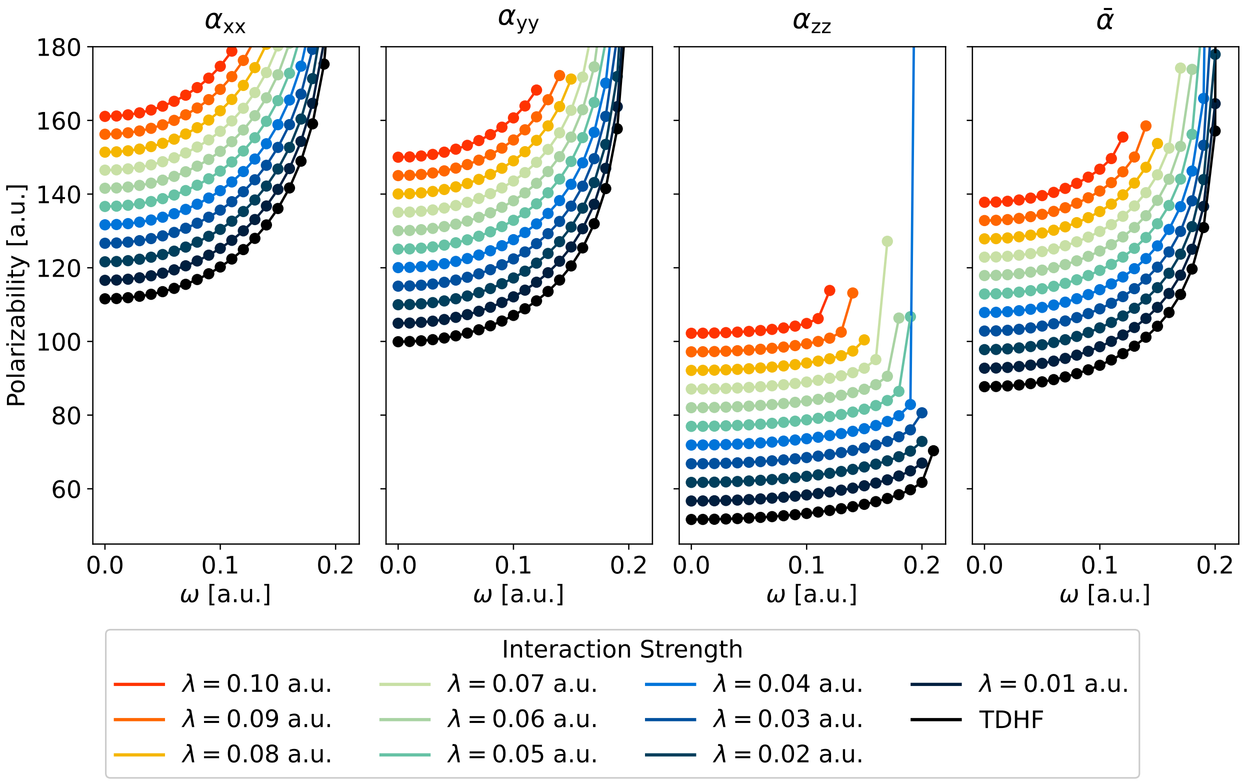

Figure S34: Dispersion curve of the frequency-dependent electric dipole polarizability of adenine in the first resonance region. Each dispersion curve is vertically offset by 5 a.u. relative to the previous one for comparison purposes.

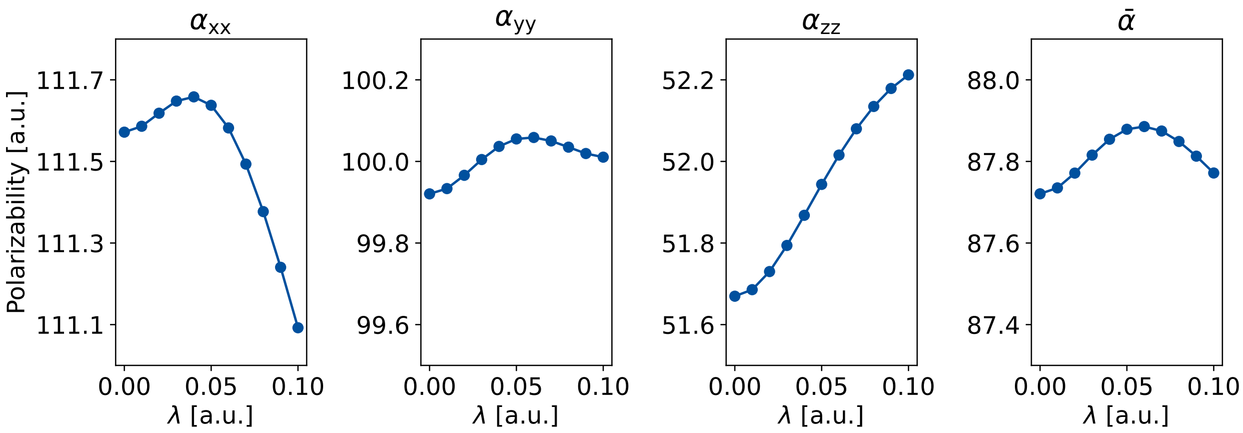

Figure S35: Static electric dipole polarizability of adenine as a function of the interaction strength.

S5.7 Guanine

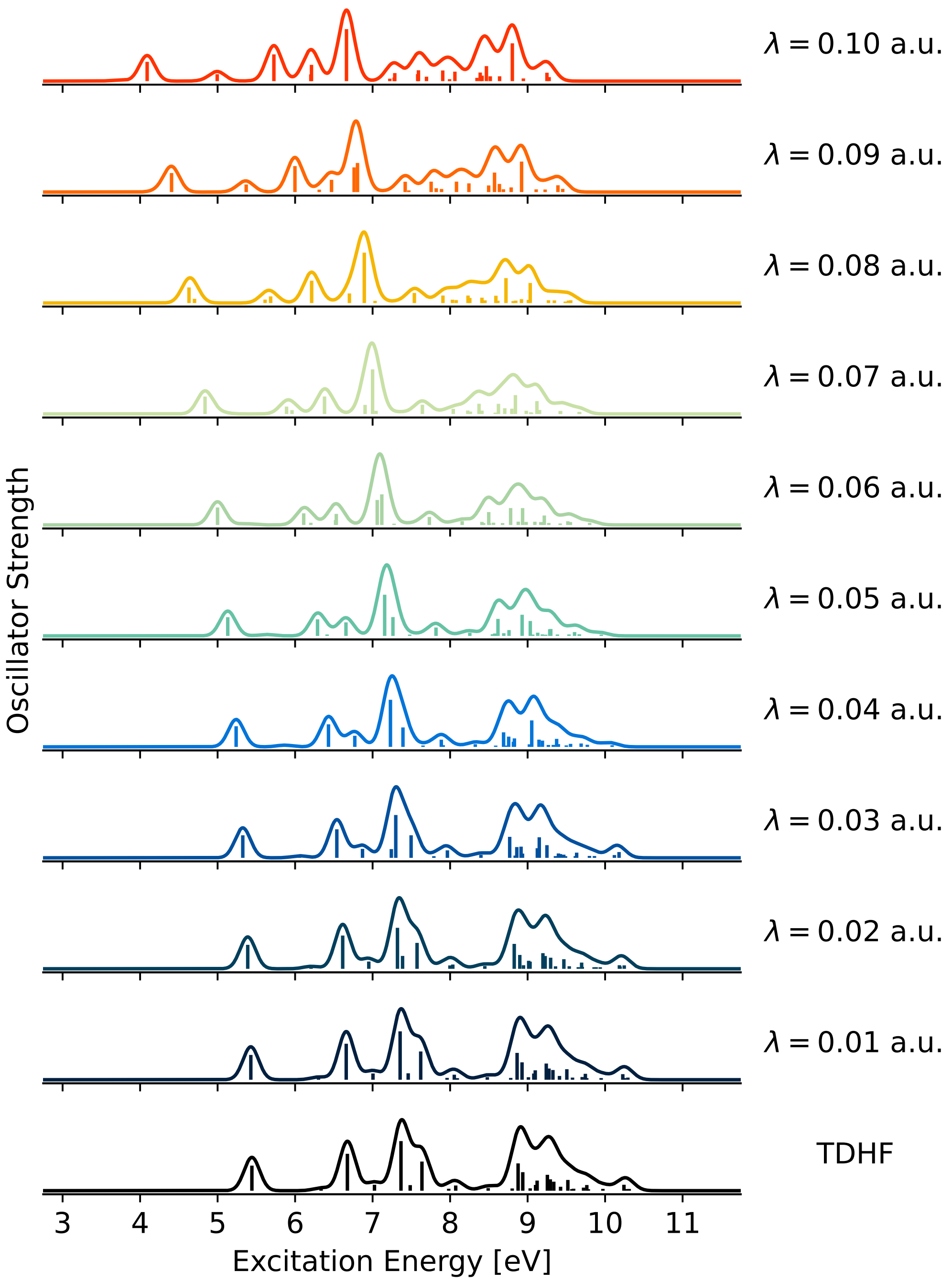

Figure S36: Valence absorption spectrum of guanine computed with different interaction strengths. Each spectrum is constructed from 50 excitations by broadening with Gaussian functions with a standard deviation of 0.1 eV. Only sticks corresponding to $f_{\mathrm{osc}} \geq 0.01$ are visualized.

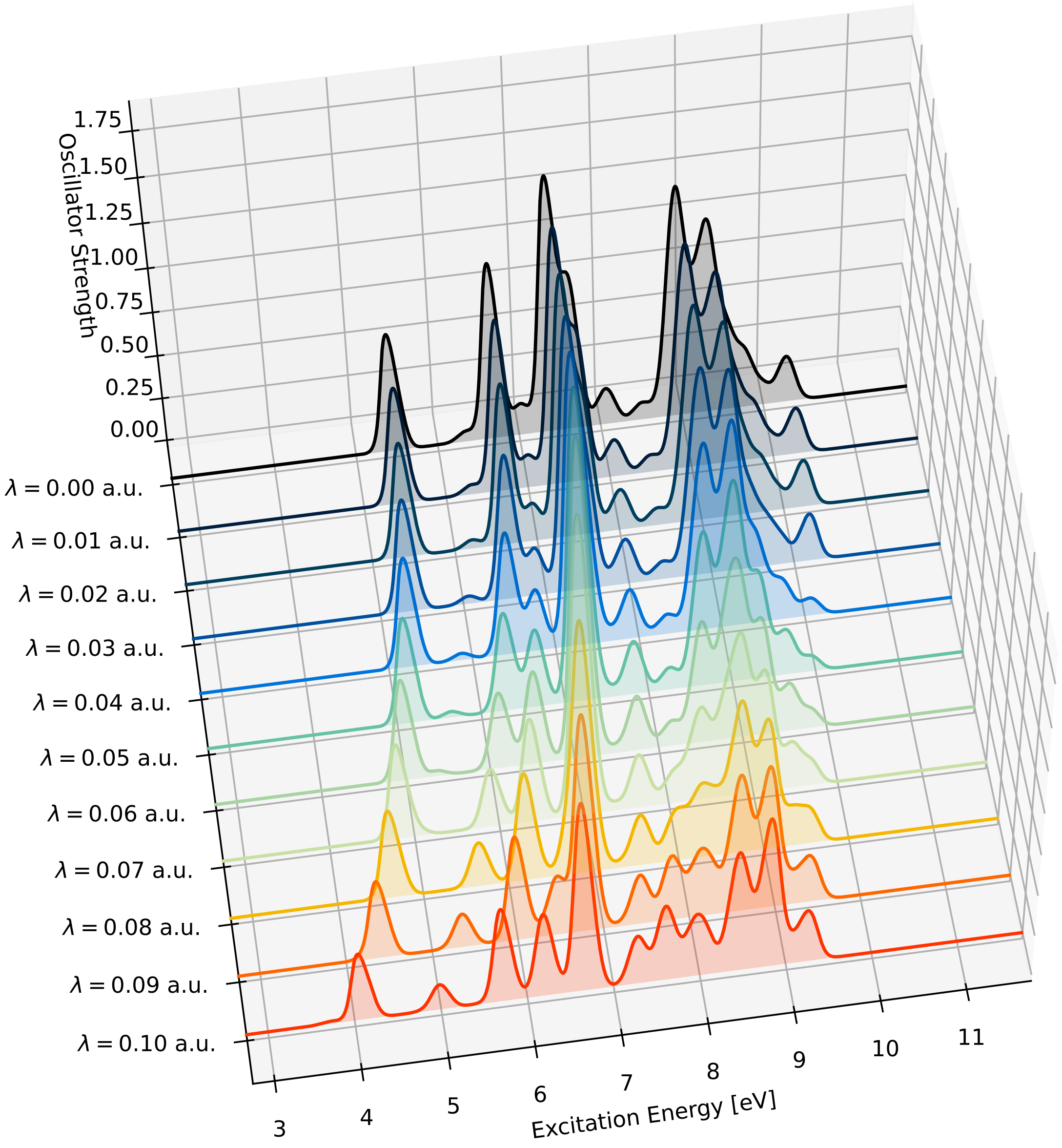

Figure S37: 3D representation of the valence absorption spectrum of guanine. Each spectrum is constructed from 50 excitations by broadening with Gaussian functions with a standard deviation of 0.1 eV.

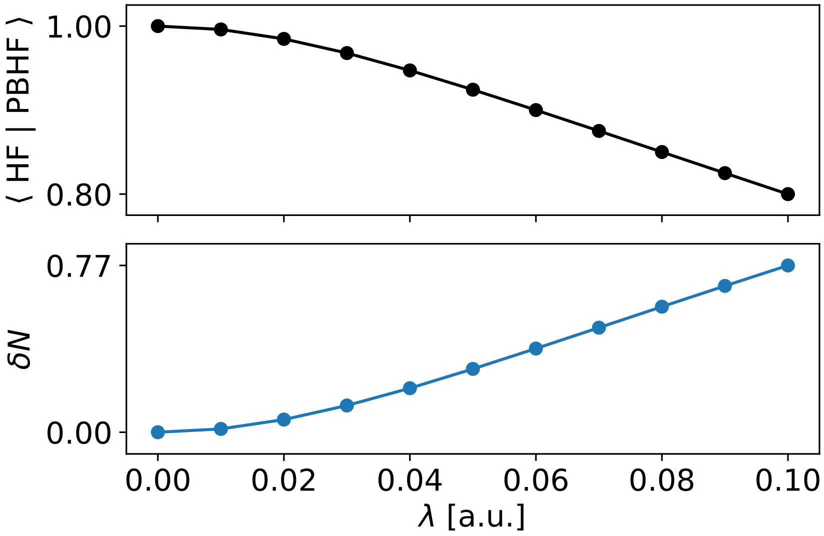

Figure S38: PBHF wave function overlap (top panel) and fractional charging (bottom panel) of guanine as a function of environmental interaction strength.

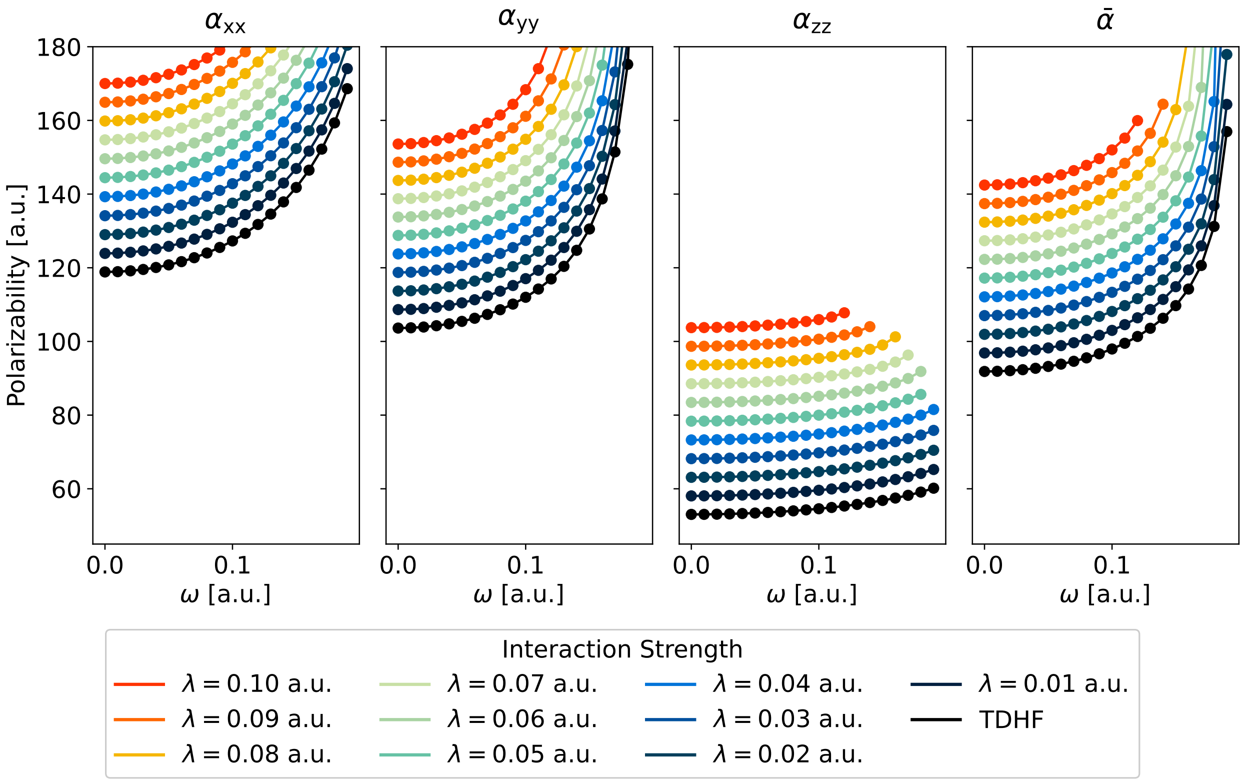

Figure S39: Dispersion curve of the frequency-dependent electric dipole polarizability of guanine in the first resonance region. Each dispersion curve is vertically offset by 5 a.u. relative to the previous one for comparison purposes.

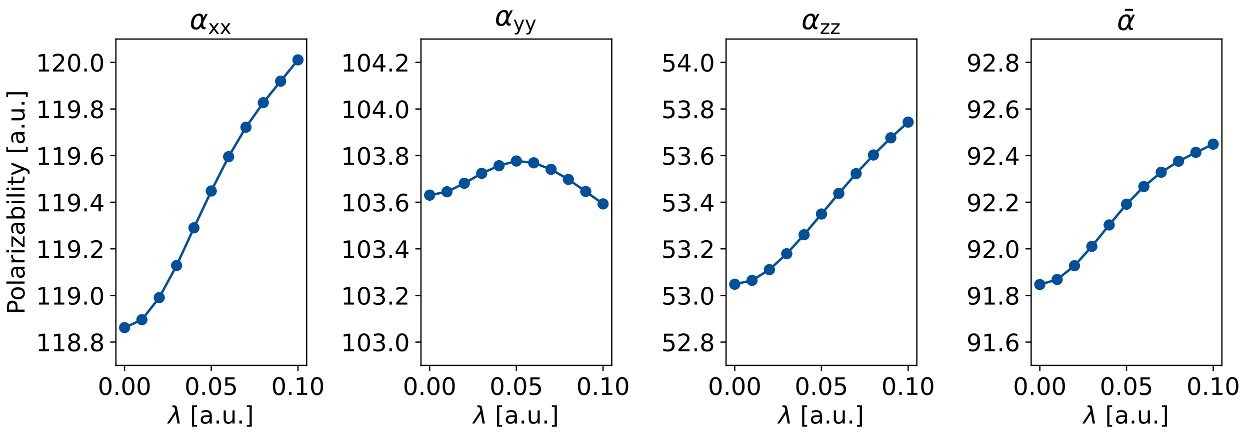

Figure S40: Static electric dipole polarizability of guanine as a function of the interaction strength.
